# Polyphosphate kinase regulates LPS structure and polymyxin resistance during starvation in *E*. *coli*

**DOI:** 10.1371/journal.pbio.3002558

**Published:** 2024-03-13

**Authors:** Kanchi Baijal, Iryna Abramchuk, Carmen M. Herrera, Thien-Fah Mah, M. Stephen Trent, Mathieu Lavallée-Adam, Michael Downey

**Affiliations:** 1 Ottawa Institute of Systems Biology, Ottawa, Ontario, Canada; 2 Department of Cellular & Molecular Medicine, University of Ottawa, Ottawa, Ontario, Canada; 3 Department of Biochemistry, Microbiology, and Immunology, University of Ottawa, Ottawa, Ontario, Canada; 4 Department of Infectious Diseases, College of Veterinary Medicine, University of Georgia, Athens, Georgia, United States of America; 5 Centre for Infection, Immunity, and Inflammation, University of Ottawa, Ottawa, Ontario, Canada; University of California, Davis, UNITED STATES

## Abstract

Polyphosphates (polyP) are chains of inorganic phosphates that can reach over 1,000 residues in length. In *Escherichia coli*, polyP is produced by the polyP kinase (PPK) and is thought to play a protective role during the response to cellular stress. However, the molecular pathways impacted by PPK activity and polyP accumulation remain poorly characterized. In this work, we used label-free mass spectrometry to study the response of bacteria that cannot produce polyP (Δ*ppk*) during starvation to identify novel pathways regulated by PPK. In response to starvation, we found 92 proteins significantly differentially expressed between wild-type and Δ*ppk* mutant cells. Wild-type cells were enriched for proteins related to amino acid biosynthesis and transport, while Δ*ppk* mutants were enriched for proteins related to translation and ribosome biogenesis, suggesting that without PPK, cells remain inappropriately primed for growth even in the absence of the required building blocks. From our data set, we were particularly interested in Arn and EptA proteins, which were down-regulated in Δ*ppk* mutants compared to wild-type controls, because they play a role in lipid A modifications linked to polymyxin resistance. Using western blotting, we confirm differential expression of these and related proteins in K-12 strains and a uropathogenic isolate, and provide evidence that this mis-regulation in Δ*ppk* cells stems from a failure to induce the BasRS two-component system during starvation. We also show that Δ*ppk* mutants unable to up-regulate Arn and EptA expression lack the respective L-Ara4N and pEtN modifications on lipid A. In line with this observation, loss of *ppk* restores polymyxin sensitivity in resistant strains carrying a constitutively active *basR* allele. Overall, we show a new role for PPK in lipid A modification during starvation and provide a rationale for targeting PPK to sensitize bacteria towards polymyxin treatment. We further anticipate that our proteomics work will provide an important resource for researchers interested in the diverse pathways impacted by PPK.

## Abstract 2

Polyphosphates are chains of inorganic phosphates that can reach over 1000 residues in length, generated by polyphosphate kinase (PPK). This study in E. coli reveals a novel role for PPK in the expression of EptA and Arn during starvation; these enzymes are required for conserved lipid A modifications that promote resistance to the last-resort antibiotic polymyxin.

## Introduction

Polyphosphates (polyP) are homopolymers of inorganic phosphates joined together by high energy phosphoanhydride bonds. Although polyP is found across diverse organisms from bacteria to humans, the intracellular concentrations, and mechanisms by which it is produced vary widely [1–3]. In *Escherichia coli* (*E*. *coli*), polyP is synthesized by the polyphosphate kinase (PPK) and degraded by the exopolyphosphatase PPX [1]. In general, *E*. *coli* produce little to no detectable polyP when undergoing logarithmic growth in nutrient rich media [4]. However, in response to diverse cellular stressors including oxidative stress caused by exposure to hypochlorous acid (bleach) [5], heat shock [6], and nutrient starvation [7], PPK rapidly synthesizes polyP using ATP as a co-substrate [1]. This stress-induced population of polyP has been linked to protein folding and turnover [5,6], transcriptional [8,9] and translational control [10], and the regulation of bacterial heterochromatin [11]. In some cases, polyP is thought to impart these changes by interacting directly with protein targets to modulate their activity. For example, polyP produced during nutrient downshift interacts with the Lon protease to direct its activity towards degradation of ADP-bound DnaA and ribosomal proteins [12–14]. Collectively, these pathways inhibit DNA replication, while increasing the intracellular pool of amino acids to help *E*. *coli* adapt to changing conditions [12,14]. PolyP can also function by chelating cations, for example, as an inhibitor of the Fenton reaction in which iron catalyzes the formation of reactive oxygen species [15]. *E*. *coli ppk* mutants (e.g., Δ*ppk*) display increased sensitivity to cellular stress [5,6,16] and decreased motility [17], biofilm formation [16], and virulence [18–20]. While the molecular events underlying these phenotypes are not always known, the role of PPK enzymes as regulators of survival during cell stress is conserved across the bacterial kingdom. Notably, in addition to synthesizing polyP, PPK enzymes can also use polyP as a donor substrate to catalyze the phosphorylation of nucleoside diphosphates [21], although the degree to which these functions contribute to stress resistance is unclear.

The PPK status of pathogenic *E*. *coli* is an important regulator of infectivity in mouse models of infection [18,22]. It has been suggested that polyP released by *E*. *coli* may play an important role in the reprogramming of macrophages, and this may involve polyP interaction with host receptors on the cell membrane or entry into host cells [18]. PPK has also been proposed as a novel target for various bacterial infections [16,23]. It is noteworthy that mesalamine, a drug used to treat ulcerative colitis and Crohn’s disease, inhibits PPK enzymes in vitro and can reduce ampicillin-resistant persister cell formation of uropathogenic *E*. *coli* in a *ppk*-dependent manner [16]. The pursuit of PPK as a valid therapeutic target demands a thorough understanding of how PPK impacts bacterial stress responses at a systems-wide level.

To better understand the role of PPK and polyP in bacterial stress responses, we used label-free proteomics to identify proteins up- or down-regulated in Δ*ppk* mutant cells relative to wild-type MG1655 K-12 controls undergoing prolonged starvation, when polyP levels are high. We report that mutant Δ*ppk* cells fail to up-regulate pathways required for amino acid biosynthesis and instead are enriched for processes related to ribosome biogenesis. In follow up work, we show a role for PPK in the modification of lipid A—the lipid anchor of the lipopolysaccharide (LPS) at the cell surface of gram-negative bacteria [24]. We demonstrate that during starvation, PPK is required for expression of EptA and the Arn proteins, and their respective phosphoethanolamine (pEtN) and 4-amino-4-deoxy-L-arabinose (L-Ara4N) lipid A modifications, as well as for expression of the upstream BasRS two-component system. In an antibiotic-resistant strain background, cells lacking *ppk* display increased susceptibility to the cationic antimicrobial peptide polymyxin B. Together, our work provides a novel resource for investigating molecular functions of polyP and new insights into how PPK inhibition might be best exploited in the clinic.

## Results

### Proteomic differences between wild-type controls and Δ*ppk E*. *coli* upon nutrient starvation

**Fig 1:**
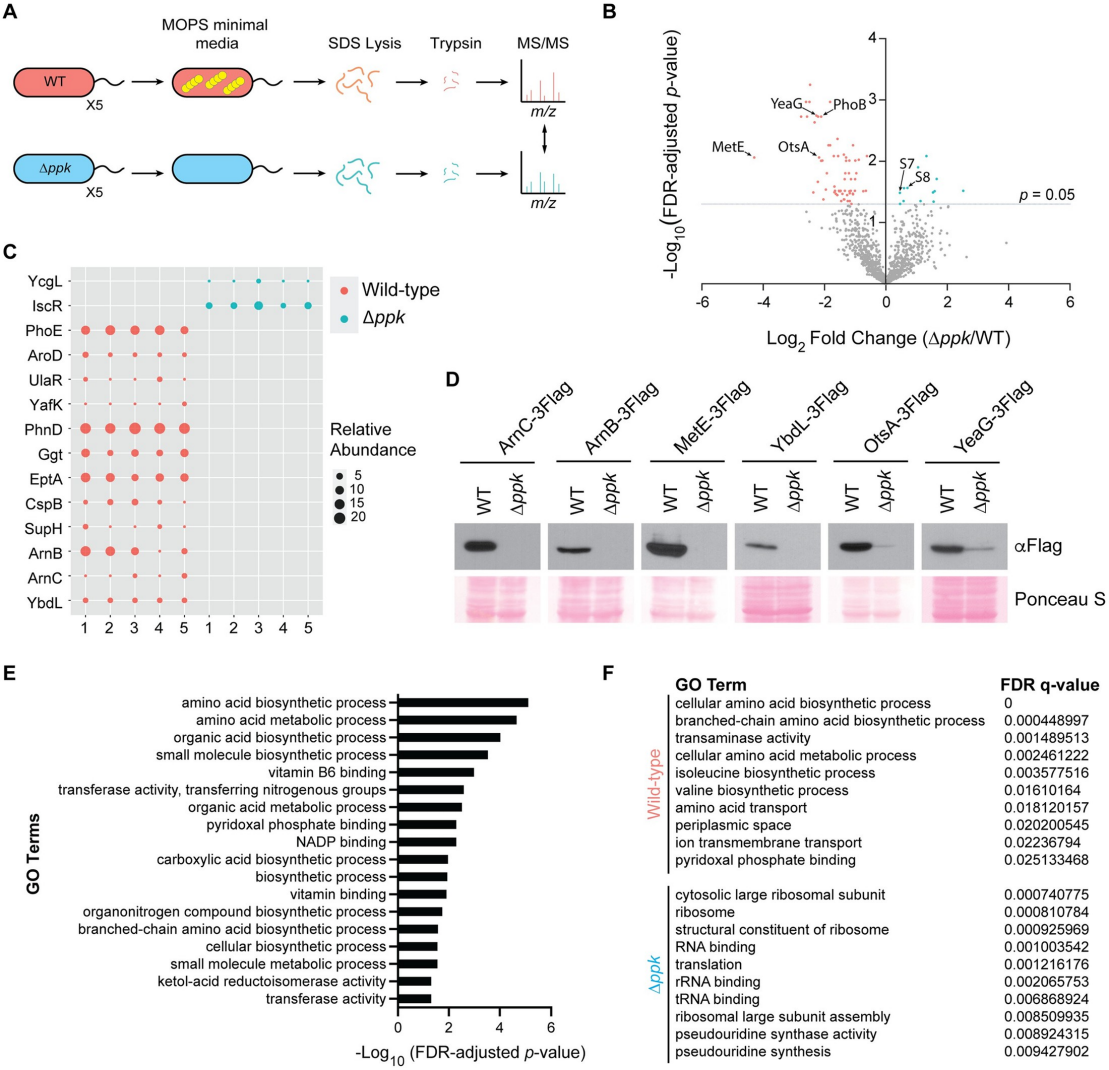
Broad proteomic changes in Δ*ppk* cells during stress. **(A)** Experimental set up for proteomics analysis. Cells were grown in LB media to mid-exponential phase before a shift into MOPS minimal media (0.1 mM K_2_HPO_4_, 0.4% glucose) for 3 h to induce amino acid starvation and polyP accumulation. The experiment was conducted using *n* = 5 biological replicates. **(B)** Volcano plot of significantly differentially expressed proteins (log_2_(fold-change Δ*ppk/*WT)). In red and blue are the significantly up-regulated proteins (FDR-adjusted *p*-value < 0.05) in wild-type and Δ*ppk* strains, respectively. **(C)** Bubble plot showing the “all-or-none” proteins detected only in either wild-type cells or Δ*ppk* mutants. Data represent the raw protein spectral counts in 5 biological replicates from each condition. **(D)** Select confirmations of mass spectrometry data. Chromosomally C-terminal 3Flag-tagged strains were grown under the same conditions used for the mass spectrometry analysis. Protein extracts were resolved using a 12% (for YbdL-3Flag) and 10% (for all other proteins) SDS-PAGE gel, transferred to PVDF membrane, and probed using an anti-Flag antibody. Images are representative of results from ≥3 experiments. **(E)** GO terms that are significantly enriched among the differentially expressed and “all-or-none proteins” identified by mass spectrometry analysis. **(F)** GO terms deemed differentially expressed based on GSEA for wild-type and Δ*ppk* mutant cells. The underlying data for Fig 1B, 1C, and 1E can be found in S1 Data. FDR, false discovery rate; GO, Gene Ontology; GSEA, Gene Set Enrichment Analysis.

We used label-free mass spectrometry analysis to compare proteomic differences between wild-type MG1655 K-12 and Δ*ppk* mutants following a shift from LB to MOPS minimal media (Fig 1A). In the bacterial polyP field, a shift from nutrient rich to MOPS minimal media is commonly used to trigger polyP accumulation [4,25,26]. At the 3-h time point used for analysis, all 5 replicates of wild-type cells showed accumulation of polyP, whereas Δ*ppk* mutants did not (S1 Fig). Bioinformatics analysis of mass spectrometry data uncovered 1,909 proteins total, of which 78 were significantly differentially expressed between the 2 conditions (false discovery rate (FDR)-adjusted *p*-value < 0.05) (Fig 1B and S1 Table). In addition, 14 proteins were classified as all-or-none (detected in 0 replicates of Δ*ppk* mutant cells but detected in all 5 replicates of wild-type cells, or vice versa) (Fig 1C and S1 Table). We used western blotting to confirm expression differences for 6 (ArnB, ArnC, MetE, YbdL, YeaG, and OtsA) out of the 7 top hits, validating the overall high-quality of the data set (Fig 1D). Only RaiA-3Flag failed to confirm in targeted western blotting experiments, showing inconsistent results between replicates.

Previous work by Varas and colleagues used mass spectrometry to compare proteomes of wild-type controls and Δ*ppk* mutants in nutrient-rich LB media [27], where there is no detectable polyP accumulation [4,28]. There, the authors identified 60 proteins up-regulated and 32 proteins down-regulated in Δ*ppk* cells [29]. The overlap between these differentially expressed proteins and the data set described in our work is poor (S2 Table). This suggests that there are vast proteomic differences between bacteria experiencing stress compared to those grown in LB media, and that there are unique roles of PPK and polyP in proteomic regulation during starvation that are uncovered by our work.

We performed Gene Ontology (GO) [30] enrichment analysis on the significantly differentially expressed and all-or-none proteins (92 total) and identified 16 enriched GO terms (FDR-adjusted *p*-value < 0.05) (Fig 1E). These included terms related to amino acid, organic acid, and small molecule biosynthesis. We next used Gene Set Enrichment Analysis (GSEA) [31] on the entire data set of 1,909 proteins to look for GO terms that are differentially expressed between the wild-type cells and Δ*ppk* mutants. This analysis showed that wild-type cells were enriched for proteins related to amino acid biosynthesis and transport (Fig 1F). In contrast, Δ*ppk* mutant cells were enriched for proteins broadly related to ribosome biogenesis and translation (Fig 1F). We also searched our data set for key regulators of the stringent response, a stress signaling system activated by nutrient starvation and mediated by the alarmones guanosine tetraphosphate (ppGpp) and guanosine pentaphosphate (pppGpp), collectively referred to as (p)ppGpp [32]. Notably, we did not observe significant differential expression of the proteins involved in (p)ppGpp synthesis such as RelA, SpoT, and GppA [33], or other regulators such as DksA [34] and RplK [35] between wild-type and Δ*ppk* mutants. Regardless, these data point to a model wherein Δ*ppk* mutants fail to properly respond to starvation by remaining primed for growth while failing to activate pathways to increase the availability of amino acids and other biomolecules needed for that purpose. In line with this interpretation, polyP interacts with the Lon protease to promote degradation of ribosomal proteins including S2, L9, and L13, as well as nucleoid proteins such as HupA, HimA (IhfA), and translational elongational protein InfC [12,36]. This degradation has been proposed to provide free amino acids to allow for targeted translation during starvation [12]. In agreement with this data, we detected significant up-regulation of 30S ribosomal proteins S7 and S8 in Δ*ppk* mutants compared to wild-type controls (Fig 1B and S1 Table). Overall, our work demonstrates that PPK is required for a timely response to MOPS-induced starvation.

### Lack of amino acids does not explain Δ*ppk* mutant phenotypes

**Fig 2:**
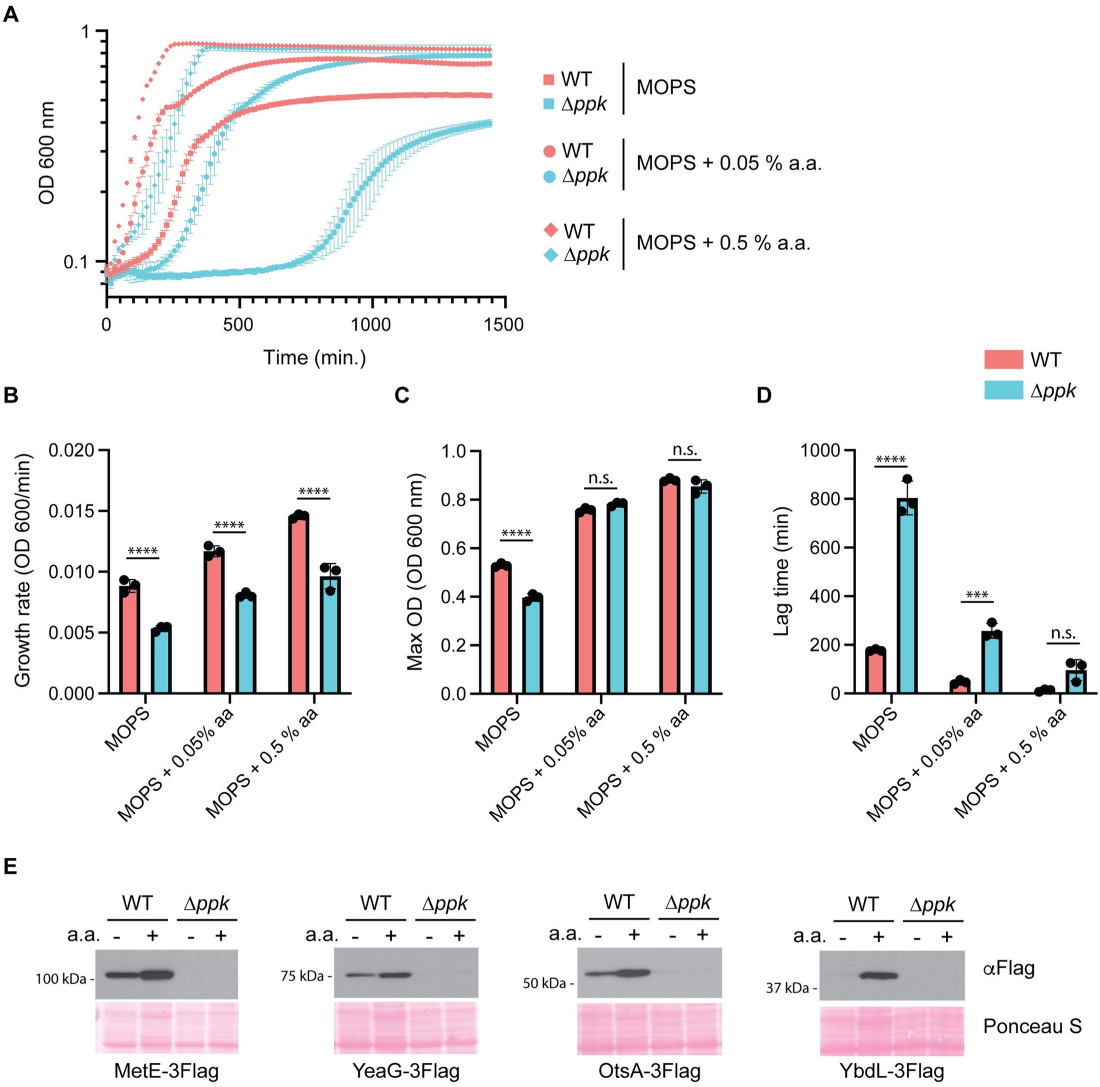
Impact of amino acid deficiencies on growth and proteome regulation in Δ*ppk* mutants. **(A)** Growth of wild-type and Δ*ppk* mutant cells in MOPS media without and with 0.05% or 0.5% amino acid supplementation. Cells were grown in LB to mid-exponential phase and then diluted to 0.1 OD_600_ in MOPS minimal media in the absence or presence of 0.05% or 0.5% amino acids. Growth was monitored using the BioscreenC plate reader (37°C with shaking, wavelength 600 nm). Error bars represent standard deviation of the mean for 3 biological replicates. Error bars not shown for data points where standard deviation is smaller than size of the symbol itself. **(B–D)** Impact of amino acids on wild-type and Δ*ppk* mutant growth dynamics. Growth rate (OD_600_/min) (B), max OD_600_ (C), and lag time (min) (D) measurements of the growth curves shown in Fig 2A were calculated using GrowthRates 6.2 (Bellingham Research Institute). Mean values with standard deviation are shown. ****, *p* < 0.0001; *** *p* < 0.001, n.s., nonsignificant via two-way ANOVA with Tukey’s post hoc analysis. The underlying data for Figs 2A–D can be found in S2 Data. **(E)** Effect of amino acid supplementation on the expression of significantly differentially expressed proteins. Cells were grown to mid-exponential phase in LB and then shifted to MOPS minimal media in the presence or absence of 0.05% amino acids for 3 h. Protein extracts were resolved using a 12% SDS-PAGE gel, transferred to PVDF membrane, and detected using an anti-Flag antibody. Images are representative of results from ≥3 experiments.

We detected up-regulation of many amino acid biosynthesis and binding enzymes in wild-type controls compared to Δ*ppk* mutants (33% of significantly differentially expressed proteins, S1 Table). We wondered if a lack of available amino acids could account for the phenotypic and proteomic differences observed in our experiments. In comparison to wild-type cells grown in MOPS media, Δ*ppk* mutants displayed decreased growth rate and maximum cell density (Fig 2A–2C). We found that Δ*ppk* cells also had a dramatic increase in the lag phase (Fig 2D). Supplementation of MOPS media with 0.05% amino acids improved the growth parameters of both strains, but the difference in growth rate and lag phase persisted (Fig 2B and 2D). The growth rate defect conferred by *ppk* mutation persisted even with the addition of 10-fold excess amino acids (0.5%) (Fig 2B). At the protein level, addition of amino acids to MOPS media increased the expression of YbdL-3Flag, with minor increases for MetE-3Flag, YeaG-3Flag, and OtsA-3Flag in wild-type cells (Fig 2E). However, in Δ*ppk* mutants, addition of amino acids failed to rescue protein expression to levels seen in untreated wild-type cells (Fig 2E). Together, these data suggest that while amino acid deficiencies may contribute to some phenotypes of Δ*ppk* mutant cells, they are unlikely to explain the broad protein dysregulation observed in our proteomics data set. Instead, we postulate that wild-type cells respond to MOPS-induced stress more promptly than Δ*ppk* cells by modulating the expression of multiple pathways. Protein differences could stem from changes in transcription or translation, or from changes in protein stability. Investigation of these distinct pathways will uncover new insights into PPK and polyP modes of action.

### PPK plays a role in regulating expression of proteins required for lipid A modification

**Fig 3:**
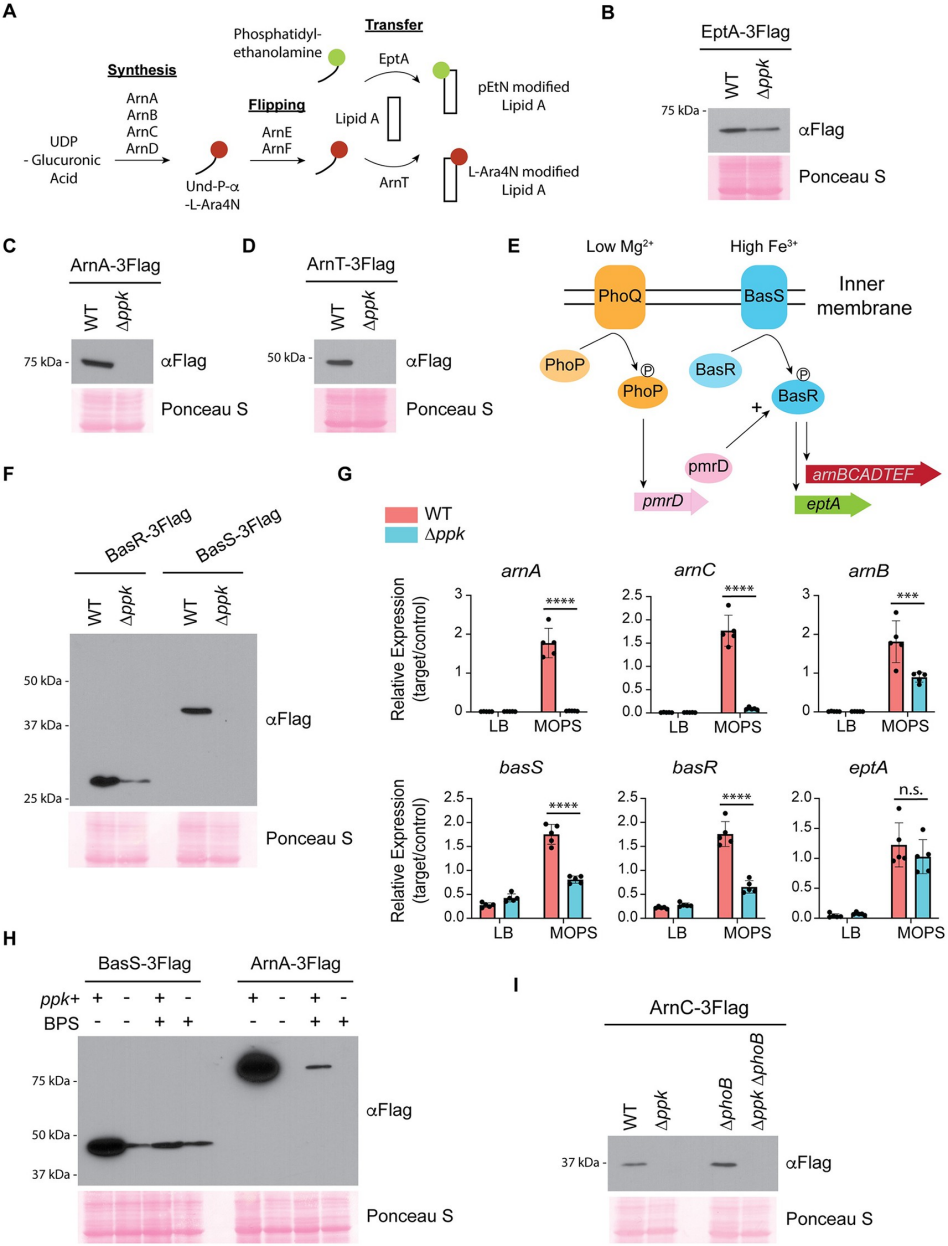
**(A) PPK positively regulates the BasRS transcriptional circuit during starvation. (A)** Schematic for Arn and EptA-catalyzed lipid A modifications in *E*. *coli*. ArnA, ArnB, ArnC, ArnD synthesize the donor substrate (Und-P-α-LAra4N). ArnE and ArnF flip and transport the donor substrate to ArnT. ArnT and EptA transfer their respective modifications to newly synthesized lipid A molecules. Note that pEtN and L-Ara4N are shown as single modifications, but doubly modified species containing 2 moieties (total) of pEtN and/or L-Ara4N are also possible. **(B–D**) Expression of EptA-3Flag (B), ArnA-3Flag (C), and ArnT-3Flag (D) following MOPS starvation for 3 h. Extracted protein samples were resolved using SDS-PAGE, transferred to PVDF, and detected using an anti-Flag antibody. Note that polar effects due to tagging may influence *eptA* regulation and visualized expression in both wild-type and mutant strain backgrounds. **(E)** Schematic showing magnesium (Mg^2+^) and iron (Fe^3+^)-dependent induction of *arnBCADTEF* and *eptA* transcription by the PhoPQ and BasRS two-component systems. **(F)** BasS-3Flag and BasR-3Flag expression following MOPS starvation. Proteins were extracted from the indicated strains and analyzed as described above. **(G)** RT-qPCR measurements of *arn*, *eptA*, and *bas* genes in wild-type and Δ*ppk* mutants during growth in MOPS minimal media. RT-qPCR analysis was conducted on cells grown to mid-exponential phase in LB followed by 3 h in MOPS minimal media. Primers and primer efficiencies used for qPCR are listed in S3 Table. Mean values with standard deviation are shown. ****, *p* < 0.0001; *** *p* < 0.001, n.s., nonsignificant via two-way ANOVA with Tukey’s post hoc analysis. The underlying data for Fig 3G can be found in S3 Data. **(H)** Role of iron in BasS-3Flag and ArnA-3Flag expression. BPS was used to chelate iron from MOPS media, following the switch from LB. At the 3-h time point, proteins were extracted and analyzed as described above. **(I)** Expression of ArnC-3Flag by Δ*phoB* mutants. Following MOPS starvation, proteins were extracted from the indicated strains and analyzed as described above. Images shown are representative of results from ≥3 experiments. PPK, polyphosphate kinase; RT-qPCR, reverse transcriptase quantitative PCR.

We were intrigued by the proteins ArnB, ArnC, and EptA, which were only detected in wild-type control but not in Δ*ppk* mutant samples, because they function in pathways associated with cationic antibiotic resistance [24,37–39]. The Arn proteins (ArnA, B, C, and D) synthesize the donor substrate for the L-Ara4N modification, undecaprenyl-phosphate-L-Ara4N, on the cytoplasmic side of the inner membrane [38–40] (Fig 3A). The undecaprenyl substrate is then flipped across the membrane by ArnE and ArnF [41] (Fig 3A). The glycosyl transferase ArnT then transfers L-Ara4N from the undecaprenyl donor to the lipid A domain of the LPS at the periplasmic face of the inner membrane [42] (Fig 3A). Like ArnT, the active site of EptA that is responsible for pEtN modification resides in the periplasm [43] (Fig 3A). Lastly, both pEtN and L-Ara4N modified lipid A are transported to the outer membrane by the LPS transport system [44]. For more information on the pathway, see review by Whitfield and Trent [45].

Together, L-Ara4N and pEtN modifications decrease the net negative charge of the outer membrane and reduce the interaction with cationic antimicrobial peptides such as polymyxin [46]. As we found for ArnC-3Flag and ArnB-3Flag in Fig 1D, expression of EptA-3Flag was reduced in *ppk* mutants compared to wild-type controls (Fig 3B). Other Arn proteins were either not detected by mass spectrometry or did not meet the stringent cut offs to allow analysis by *t* test (i.e., Fig 1B), but we used western blotting to confirm the same trend for ArnA-3Flag and ArnT-3Flag (Fig 3C and 3D). Importantly, Arn protein levels could be restored by plasmid-based expression of *ppk* from its endogenous promoter (pPPK, S2 Fig). In fact, cells expressing pPPK in either wild-type or Δ*ppk* mutant backgrounds had somewhat higher levels of Arn proteins compared to wild-type strains with empty vector controls (S2 Fig). We surmise that PPK is somewhat overexpressed in both strain backgrounds due to multiple copies of the plasmid and that Arn expression scales with total PPK levels.

Next, we asked if the switch from LB to MOPS media was triggering Arn expression. As anticipated, Arn proteins were low for both wild-type and Δ*ppk* mutants during growth in LB, and it was only during prolonged growth in MOPS that their levels increased in wild-type cells (S3A Fig). This led us to check if regulation depended on the BasRS two-component system, which sits upstream of the *arnBCADTEF* operon and *eptA* gene, and responds to various stresses (Fig 3E). In *E*. *coli*, the BasS membrane protein auto-phosphorylates in response to high concentrations of iron and zinc, and subsequently trans-phosphorylates the transcription factor BasR to promote transcription of *arnBCADTEF* and of *eptA*, which is found in the same operon as *basS* and *basR* [47–50]. In parallel, the PhoPQ two-component system, activated under conditions of low magnesium promotes BasR activation via PmrD (Fig 3E) [51,52]. We found that expression of both BasS-3Flag and BasR-3Flag were decreased in Δ*ppk* mutant cells, compared to wild-type cells, during starvation (Fig 3F). In contrast, we found that PhoP and PhoQ-3Flag protein levels remained largely unchanged (S3B and S3C Fig), and the induction of ArnC-3Flag expression was not changed by the inclusion of excess magnesium (S3D Fig). Thus, while we cannot rule out additional points of regulation, our data support a model wherein Arn and EptA expression during starvation in MOPS depends on upstream PPK and/or polyP-dependent regulation of the BasRS two-component system. Indeed, qPCR analysis demonstrated that MOPS treatment induced the transcription of Arn, BasS, and BasR encoding genes in wild-type cells, and this response was defective in Δ*ppk* mutants (Fig 3G). Interestingly, loss of *ppk* had a less dramatic impact on *arnB* expression than that of *arnA* and *arnC*, even though they are encoded in the same operon (Fig 3G). Likewise, we did not observe a significant difference between wild-type and *ppk* mutants for *eptA* expression (Fig 3G), despite its regulation at the protein level. This suggests the possibility that PPK and/or polyP exert their function at multiple levels. Importantly, differences in expression of genes within a single operon have been documented previously and could stem from variations in transcription initiation from additional promoter elements or mRNA processing [53]. Finally, there are several reports of BasR and BasS regulation by the stationary phase sigma factor RpoS [54,55]. However, we found that Δ*ppk* mutants had RpoS protein levels that were similar to those of wild-type cells during starvation in MOPS (S3E Fig), suggesting additional modes of action.

To our knowledge, this is the first description of *E*. *coli* EptA and Arn protein induction by MOPS media, and the mechanism at play is unknown. In LB media, the BasRS transcriptional circuit induces Arn expression and downstream modifications in the presence of high iron levels (>200 μm) [47,56], but the iron concentration in MOPS is quite low (10 μm). Still, we remained curious about the role of iron based on a previous report that MOPS-induced polyP can bind iron to inhibit the Fenton reaction, which decreases the production of reactive oxygen species [15]. Consistent with a requirement for iron in BasS activation, treatment with iron chelator BPS blunted expression of Arn-3Flag proteins in wild-type cells grown in MOPS (Fig 3H). However, we note that loss of *ppk* did not impact expression of Arn-3Flag proteins in LB media treated with high iron (S3F Fig). Thus, while iron is important for BasRS induction in both LB and MOPS, the impact of Δ*ppk* is unique to MOPS. We reasoned that BasRS regulation by PPK could depend on polyP accumulation, which occurs in MOPS (S1A Fig), but not in LB treated with iron (S3G Fig). To test this idea, we analyzed Arn protein induction in Δ*phoB* mutants, which are deficient in polyP accumulation even in MOPS media (S3H Fig) [4,25]. Additionally, PhoB is down-regulated in Δ*ppk* mutants compared to wild-type cells (Fig 1B and S1 Table). To our surprise, Δ*phoB* cells had levels of Arn-3Flag expression comparable to wild-type controls (Fig 3I), suggesting that additional PPK activities beyond polyP synthesis may contribute to the molecular phenotypes described here (see Discussion).

### pEtN and L-Ara4N modifications are down-regulated in Δ*ppk* mutants

**Fig 4:**
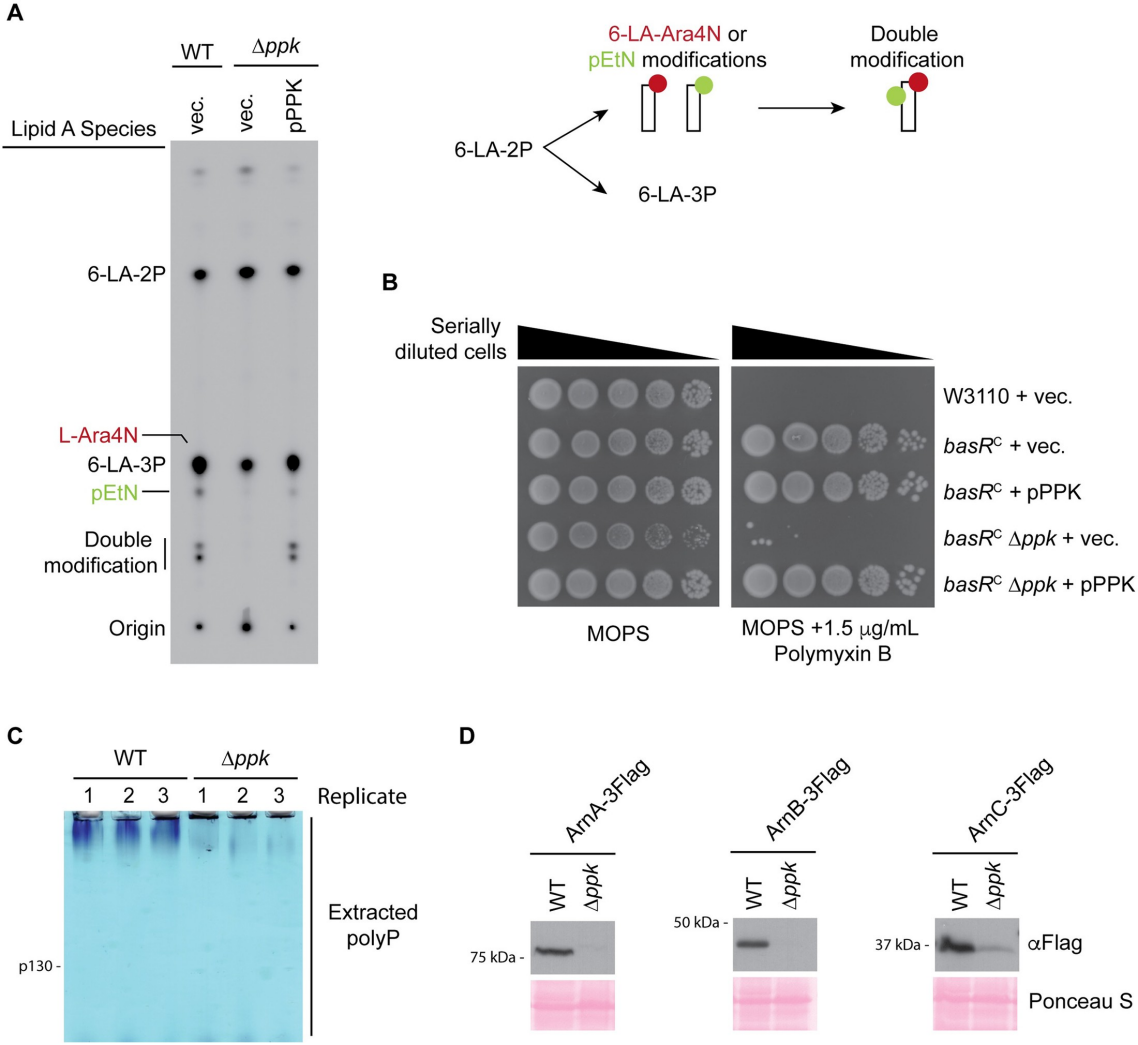
Consequences of LPS mis-regulation in *ppk* mutants. **(A)** Schematic of lipid A modification (right) and lipid A profiles (left) of strains mutated for or expressing *ppk*. The indicated strains were grown in LB until mid-log phase before being shifted to MOPS media supplemented with ^**32**^P for 6 h. Lipid A was isolated as described in the Materials and methods and analyzed via TLC prior to phosphorimaging. ^**32**^P-labeled lipid A species are labeled on the left side of the image. During growth in LB, most of the lipid A (LA) is hexa-acylated and *bis*-phosphorylated (6-LA-2P) and ~1/3^rd^ is modified with an additional phosphate group (6-LA-3P). Upon stimulation of BasRS, 6LA-2P is used as the substrate for EptA and ArnT to generate pEtN and L-Ara4N-modified lipid A species. Note that the singly modified 6-LA-Ara4N species is not resolved from the 6-LA-3P species. “Doubly-modified” refers to lipid A species carrying 2 moieties (total) of pEtN and/or L-Ara4N. Images shown are representative of results from ≥3 biological replicates. **(B)** Polymyxin growth phenotypes of Δ*ppk* mutants. The indicated strains were spotted in 10-fold serial dilutions on the indicated media and incubated at 37°C for 2 days prior to imaging. Images shown are representative of results from ≥3 biological replicates. **(C)** PolyP extracts from wild-type and Δ*ppk* mutant UPEC strains. Cells were grown in LB media to mid-exponential phase and then switched to MOPS minimal media for 3 h. PolyP was extracted from the indicated strains and analyzed on a TBE-urea gel stained with toluidine blue. The migration of a chain ~130 phosphate residues in length (p130) is indicated. **(D)** PPK-dependent ArnA-3Flag, ArnB-3Flag, and ArnC-3Flag expression in UPEC. Cells were grown as described in C. Extracted protein samples were resolved using SDS-PAGE, transferred to PVDF, and detected using an anti-Flag antibody. Images shown are representative of results from ≥3 experiments. LPS, lipopolysaccharide; TLC, thin-layer chromatography.

Next, we directly examined lipid A modifications that depend on Arn and EptA expression, namely L-Ara4N and pEtN addition. For these experiments, we used the W3110 strain (K-12) background that has been used extensively for lipid A analyses [57,58]. As a control, these strains showed PPK-dependent expression of Arn-3Flag proteins in MOPS media, similar to what we observed in the MG1655 background used for our other assays (S4A Fig). A time course of protein expression showed that in wild-type cells, ArnC-3Flag was detectable after 3 h in MOPS media and remained up-regulated for the duration of the experiment (S4B Fig). In contrast, ArnC-3Flag was undetectable in Δ*ppk* mutants after 6 h in MOPS media and was only observed after overnight growth (S4B Fig). Therefore, we chose 6 h (3 h post-Arn up-regulation) as a time point to measure lipid A modifications using radio-labeling and thin-layer chromatography (TLC). Indeed, compared to their wild-type counterparts, we saw that Δ*ppk* mutants were defective in the accumulation of lipid A species singly or doubly modified with pEtN and L-Ara4N modifications (Fig 4A). This defect was fully rescued by introduction of the pPPK plasmid (Fig 4A). Therefore, differences in levels of Arn and EptA proteins translate to changes in lipid A modification between wild-type and Δ*ppk* mutants.

### PPK promotes polymyxin resistance

Positively charged L-Ara4N and pEtN modifications play a role in resistance to cationic antimicrobial peptides that alter membrane permeability and structure [59–61]. To study the phenotypic consequences of disrupting PPK-dependent regulation of lipid A modifications, we focused on polymyxin antibiotics, used both topically to treat gram-negative bacterial infections, and systemically as a last-resort antibiotic in the clinic [62]. For these experiments, we used a polymyxin resistant strain (WD101) that carries a constitutively active *basR* allele (*basR*^*C*^) resulting in lipid A that is heavily modified with L-Ara4N and pEtN [63]. This strain is otherwise isogenic to W3110 [63]. Importantly, compared to wild-type cells, Arn-3Flag protein expression was decreased in Δ*ppk* mutants carrying this allele as observed previously in other backgrounds (S4A Fig). Using dilution assays, we confirmed that the *basR*^*C*^ strain is resistant to polymyxin compared to the wild-type W3110 counterparts grown on MOPS (S4C Fig) and this resistance requires ArnA (S4D Fig). Deletion of *ppk* decreased polymyxin resistance in the *basR*^*C*^ strain (Figs 4B and S4C), and this effect could be reversed by introduction of the pPPK plasmid (Fig 4B). These experiments demonstrate that *ppk* contributes to *basR*^C^-mediated polymyxin resistance under starvation conditions. Finally, we were interested in testing whether the role of *ppk* in Arn expression was conserved in pathogenic *E*. *coli*. For this, we used a uropathogenic *E*. *coli* (UPEC) strain (UTI89). We found that UPEC also accumulated polyphosphate during MOPS starvation (Fig 4C) and that Arn-3Flag protein expression in these strains was dependent on *ppk* (Fig 4D). We speculate that targeting PPK in UPEC may serve as a strategy to sensitize cells to drug treatment following acquisition of polymyxin resistance.

## Discussion

Our study is the first whole proteome analysis in *E*. *coli* comparing wild-type cells to Δ*ppk* mutants under conditions permissive for polyP synthesis. Our work will serve as a resource for researchers interested in how PPK and polyP control protein homeostasis, and, more broadly, how bacterial cells respond to starvation. We find that Δ*ppk* mutant cells remain poised for growth during starvation at the expense of up-regulating biosynthetic pathways for nutritional building blocks such as amino acids. We further elaborate a critical role for PPK in regulating the conserved BasRS two-component system and downstream expression of proteins involved in lipid A modifications. Finally, we uncover evidence that PPK-dependent differences in lipid A modification contribute to polymyxin sensitivity. Together, our results illuminate a previously unexplored role of PPK in lipid A modification and antibiotic resistance.

The simplest interpretation of our data, illustrated in S5 Fig, is that PPK and/or polyP promote expression or activation of the BasS sensor protein and its cognate transcription factor BasR. In turn, BasR acts to stimulate transcription of both itself and genes encoding the EptA and Arn proteins. In support of the idea that the BasRS-Arn circuit is regulated by polyP, as distinct from PPK, we only observed defects in Arn protein expression in MOPS media where polyP is synthesized to high levels. In LB media supplemented with iron, where polyP is absent, no differences in Arn protein expression was observed between wild-type strains and Δ*ppk* mutants. However, we note that a proposed role for polyP is complicated by the observation that Δ*phoB* strains express ArnC-3Flag protein at a level similar to wild-type cells, even though they are largely deficient in polyP accumulation. Based on this finding, it is possible that an unknown activity of PPK may underlie the molecular and cellular phenotypes described here. Alternatively, a nonzero level of polyP has been detected in Δ*phoB* strains previously [4], and this pool of polyP, or at least its cyclical synthesis and destruction, may be sufficient to drive Arn expression. In this scenario, polyP could act upstream and interact directly with BasS, BasR, or as-yet-unknown transcriptional regulators to promote transcription of the *eptA-basR-basS* operon.

The impact of PPK could also be several steps removed from the transcriptional activities occurring directly at *eptA-basR-basS*. Here, it is particularly relevant that the trigger for activation of the BasRS transcriptional program during MOPS starvation is unknown. The failure of wild-type cells to up-regulate Arn expression in the presence of the iron chelator BPS suggests an iron-dependent response. However, given that induction of Arn protein expression occurs abruptly after 3 h, we theorize that additional time-dependent changes are required. In media-switch experiments, we found that incubation of “spent” MOPS media from wild-type cells that have induced ArnC-3Flag expression fails to rapidly trigger ArnC-3Flag expression in either wild-type or Δ*ppk* mutants (S6A and S6B Fig). This observation argues against changes in media composition as the sole contributor to BasS activation in MOPS, and intracellular depletion of specific metabolites or accumulation of by-products is likely required. Finding the trigger for BasS activation will provide important clues into unknown functions of PPK and new insights into the regulation of lipid A modifications.

Collectively, our findings underscore that the pathways regulating modification of *E*. *coli* LPS, and the associated changes in antibiotic resistance, are intimately coupled to nutrient availability. In the context of host infection, the validity of PPK as a target to sensitize bacteria to cationic peptides like polymyxin may depend on whether bacteria at the site of infection are exposed to conditions that activate PPK. Intriguingly, there is evidence to suggest that bacteria colonizing in the center of dense biofilms experience starvation concomitant with decreased susceptibility to antibiotics [64–66]. We theorize that PPK may play an important role in this context. Notably, the immediate molecular events that activate PPK during stresses of any kind remain unclear [26,67–69]. Our work predicts that genes encoding PPK activators are also likely to play a role in polymyxin resistance.

In general, the enzymes involved in LPS modifications, including the pEtN and L-Ara4N modifications, are highly conserved across gram-negative bacteria [59]. Yet, there are important differences between some species in the details of regulation, such as in the crosstalk between two-component systems [51,70]. As such, it is imperative to test if PPK and polyP impact these modifications in other bacteria. We anticipate that comparing and contrasting the role of PPK across multiple species will help to elucidate molecular mechanisms governing antibiotic sensitivity and the acquisition of resistance.

## Materials and methods

### Bacterial strains, media, and growth

All bacterial strains and plasmids, as well as their sources, used in this work are listed in S3 Table. Reagents are listed in S4 Table.

Tagged and deletion strains were generated using lambda-red–mediated site-specific recombination via the arabinose or heat shock inducible systems expressed from pKD46 [71] and pSIM6 [72] plasmids, respectively. The kanamycin deletion and C-terminal 3Flag-kanamycin tagging cassettes were amplified from pKD4 [71] and pSUB11 [73], respectively. In select cases (S3 Table), resistance markers were excised using FLP recombinase activity expressed from pCP20 [74]. Antibiotics were added when appropriate: kanamycin (50 μg/ml) and ampicillin (100 μg/ml). For recombineering genetic transformations cells were made electrocompetent and transformed using protocols described previously [75]. Plasmids were also introduced into the bacteria by electroporation.

#### Nutrient downshift

All strains were grown in LB media at 37°C unless carrying pSIM6 and pKD46 plasmids which were grown at 30°C. For nutrient downshift experiments overnight cultures, grown in LB media, were diluted to 0.1 OD_600_ in LB the next day and grown to mid-exponential phase (approximately 0.6 OD_600_). Cells were then washed twice with 1xPBS and resuspended in MOPS minimal media (Teknova) supplemented with 0.1 mM K_2_HPO_4_, 0.4% glucose—this recipe was used for starvation unless otherwise indicated. Cells were grown in minimal media for 3 h before harvesting on ice. Cell pellets were flash frozen using dry ice and stored at −80°C.

#### Other growth conditions

Where indicated, exponentially growing LB cultures were switched to MOPS minimal media containing casamino acids (Bacto) at a final concentration of 0.05% and 0.5%. Where indicated, iron in MOPS media was chelated using 0.2 mM bathophenanthrolinedisulfonic acid disodium salt hydrate (BPS). BPS was added to MOPS media at the start of the nutrient downshift. Where indicated, LB media was supplemented with 0.2 mM iron sulfate when overnight cultures were diluted to 0.1 OD_600_. LB minus and plus iron cultures were grown to mid-exponential phase (about 1.5 h) at 37°C before harvesting cells. Where indicated, exponentially growing LB cultures were switched to MOPS minimal media containing 1 mM magnesium chloride or 1 mM calcium chloride and grown at 37°C for 3 h before harvesting.

### Mass spectrometry

#### Cell growth

Five overnight cultures (*n* = 5) were prepared for each wild-type and *ppk* strains from freshly streaked plates. The next day, overnight cultures were diluted in 100 ml LB and grown to mid-exponential phase, washed 2 times with 1xPBS, and then switched to MOPS minimal media without amino acids for 3 h as described above (methods: nutrient downshift), and 60 OD_600_ and 5 OD_600_ equivalents of cell volume were harvested for mass spectrometry analysis and polyP extraction, respectively.

#### Protein extraction and precipitation

Protein extracts were prepared by trichloroacetic acid (TCA) precipitation. Cells pellets were thawed on ice and resuspended in 700 μl mass spectrometry-grade lysis buffer (5% SDS, 100 mM tetraethylammonium bromide (TEAB), Roche cOmplete protease inhibitor cocktail tablets). The cell suspension was lysed by sonication on ice for 30 s at power level 3 with 1-min break in between, for a total of 3 times. The cell lysate was then centrifuged at 13,000 rpm, 4°C for 15 min. The supernatant was transferred to a new tube and centrifuged again for 10 min prior to being collected.

Proteins were precipitated by adding 2.8 ml 15% TCA in acetone to the cell lysate (brings final concentration of TCA to 10%), mixing by inverting and incubating at −20°C overnight. The next day, the samples were centrifuged at max speed, 4°C for 10 min and supernatant was discarded immediately after the spin. The pellet was air dried for 30 min, then resuspended in dissolution buffer (5% SDS, 100 mM TEAB) prior to BCA quantification and lyophilization. Lyophilized samples were sent to UC Davis Proteomics Core for mass spectrometry analysis.

#### Sample preparation

The following protocols (3–5) were provided by the UC Davis Proteomics Core with minor alterations.

Lyophilised proteins were solubilized in 50 μl of solubilization buffer, consisting of 5% SDS, 50 mM triethyl ammonium bicarbonate, pH 7.5. A Bichinoic Acid Assay was taken of all samples, and 100 μg total protein from each sample was then used to perform protein digestion via suspension-trap devices (S-Trap) (ProtiFi). Disulfide bonds were reduced with dithiothreitol and alkylated with iodoacetamide in 50 mM TEAB buffer. The enzymatic digestion consisted of an addition of trypsin at 1:100 enzyme: protein (wt/wt) for 4 h at 37°C, followed by a boost addition of trypsin using same wt/wt ratios for overnight digestion at 37°C. Peptides were then eluted from the S-Trap by sequential application of elution buffers of 100 mM TEAB, 0.5% formic acid, and 50% acetonitrile 0.1% formic acid. The eluted tryptic peptides were dried in a vacuum centrifuge prior to re-constitution in 0.1% trifluoroacetic acid. These were subjected to Liquid Chromatography couple to tandem Mass Spectrometry (LC-MS/MS) analysis as described below.

#### Liquid chromatography

Peptides were resolved on a Thermo Scientific Dionex UltiMate 3000 RSLC system using a PepMap 75 μm × 25 cm C18 column with 2 μm particle size (100 Å pores), heated to 40°C. A final volume of 5 μl was injected, corresponding to 1 μg of total peptide, and separation was performed in a total run time of 90 min with a flow rate of 200 μl/min with mobile phases A: water/0.1% formic acid, and B: 80%ACN/0.1% formic acid. Gradient elution was performed from 10% to 8% B over 3 min, from 8% to 46% B over 66 min, and from 46 to 99% B over 3 min, and after holding at 99% B for 2 min, down to 2% B in 0.5 min followed by equilibration for 15 min.

#### Mass spectrometry

The peptides were analyzed on an Orbitrap Fusion Lumos (Thermo Fisher Scientific) mass spectrometer. Spray voltage was set to 1.8 kV, RF lens level was set at 46%, and ion transfer tube temperature was set to 275°C. The mass spectrometer was operated in a data-dependent acquisition mode. A survey full scan mass spectra (from m/z 375 to 1,600) was acquired in the Orbitrap at a resolution of 60,000 (at 200 m/z). The automatic gain control (AGC) target for MS1 was set as 4e5, and ion filling time was set as 50 msec. The *n* = 15 most abundant precursor ions with charge state +2, +3 were isolated in a 3-s cycle, isolation window width of 1.2 m/z, fragmented by using collision-induced dissociation (CID) fragmentation with 30% normalized collision energy, and detected via IonTrap, with a scan rate set to Rapid. The AGC target for MS/MS was set as 5e3 and ion filling time was set at 35 msec. The dynamic exclusion was set to 50 s with a 10-ppm (parts per million) mass window.

### Mass spectrometry bioinformatics analysis

#### Protein identification

Mass spectrometry RAW data were processed using the Trans-Proteomic Pipeline (TPP v5.2.0) [76]. Files from the mass spectrometry runs were converted to mzML files using the msconvert tool from ProteoWizard [77] (v3.0.22088). Comet [78] (v2018.01.04) was used to search the files against the UniProt [79] *E*. *coli* protein sequence database (UP000000625 downloaded 2021-08-04), along with a target-decoy strategy where all protein sequences were reversed. The database search was performed with trypsin as a digestive enzyme, allowing for up to 3 missed cleavages and considering semi-tryptic digestion. The peptide mass tolerance was set to 20 ppm. Carbamidomethylation of cysteine was set as a fixed modification, and the variable modifications considered were deamidation of asparagine and glutamine, as well as oxidation of methionine. The probability of protein identifications was evaluated with ProteinProphet [80], and proteins identified at an FDR < 1% were deemed confidently identified.

#### Differential expression analysis

The spectral counts from confidently identified proteins were used for downstream analysis. Proteins with zero spectral counts in a given experiment were imputed a quantification value by random sampling of the lowest 20% of nonzero spectral counts in the entire data set to account for missing values. All spectral counts were normalized by the total number of spectral counts in a given experiment. Differential expression was assessed on the normalized spectral counts using a two-tailed, two-sample Student’s *t* test, assuming unequal variance. A *t* test was performed for proteins that had at least 3 nonzero spectral counts out of 5 replicates prior to imputation in both experimental conditions, and the Benjamini–Hochberg [81] procedure was used to adjust *p*-values for multiple hypothesis testing. Proteins with an FDR-adjusted *p*-value < 0.05 were considered significantly differentially expressed. Finally, proteins that were identified in all replicates of one condition and not identified in any replicates of the other condition did not undergo a *t* test, but still reflect a significant expression difference. As such, these were named “all-or-none” proteins and are reported as having differential expression.

#### Gene Ontology enrichment analysis

Ontologizer [82] (v2.0) was used to identify Gene Ontology [30] annotations that were significantly enriched in the set of differently expressed proteins (FDR-adjusted *p*-value < 0.05) and “all-or-none” proteins. The enrichment was performed against a background of all proteins identified with mass spectrometry (confidently identified at an FDR < 1%). The OBO ontology file and the GAF annotation file used in the analysis were downloaded from http://geneontology.org/ on 2023-02-13. The *p*-values were adjusted using the Benjamini–Hochberg procedure, and GO terms that were enriched with an adjusted *p*-value < 0.05 were considered significantly enriched.

#### Gene set enrichment analysis

A GSEA [31] (v4.1.0) was performed (on 2023-05-24) to identify sets of GO terms that were enriched in the entire set of proteins (confidently identified at an FDR < 1%). Gene sets were built of GO terms and their annotated proteins, and GSEA excluded GO terms that annotated more than 1,000 proteins or less than 3 proteins to remove general and highly specific terms, and 1,000 gene set permutations were used to estimate enrichment scores. The enrichment scores were normalized with the “meandiv” parameter to allow for a more accurate comparison of enrichment scores across gene sets. Gene sets with a *q*-value < 0.05 were considered significantly enriched.

### Polyphosphate extraction

#### Polyphosphate extraction

Cells were grown as indicated and polyP extraction was conducted as described previously [83]. For clarity, similar language is used to describe the protocol here. Five OD_600_ equivalents of pelleted cells were thawed on ice, resuspended in 400 μl of LETS buffer (100 mM LiCl, 10 mM EDTA, 10 mM Tris-HCl, 0.2% SDS) at 4°C, then transferred to a tube containing 600 μl of room temperature neutral phenol (pH 8) and 150 μl of RNase-free water. Tubes were vortexed for 20 s and 600 μl chloroform was added. Next, tubes were again vortexed for 20 s and then centrifuged for 2 min at 13,000 g. The top 600 μl layer was transferred to a new tube containing 600 μl of chloroform before vortexing for 20 s and centrifuging for 2 min at 13,000 g. The top 400 μl layer was transferred to a new tube and treated with 2 μl of 10 mg/ml RNaseA and DNaseI, each, for 1 h at 37°C. Next, the mixture was transferred to prechilled tubes containing 1 ml 100% ethanol and 120 mM sodium acetate (pH 5.3) and left overnight at −20°C to precipitate. The next day, samples were centrifuged for 20 min at 13,000 g for 20 min at 4°C. Supernatant was discarded and 500 μl 70% ethanol was added before centrifuging for 5 min at 13,000 g at 4°C. Supernatant was again discarded and pellet was air dried to remove trace ethanol. The translucent polyP pellet was resuspended in 30 μl sterile water and stored at −80°C.

#### Gel analysis

Extracted polyP was visualized using a 15.8% TBE-urea gel (5.25 g urea, 7.9 ml 30% acrylamide, 3 ml 5xTBE, 150 μl 10% APS, and 15 μl TEMED). Extracted polyP was mixed at a 1:1 ratio with loading dye (10 mM Tris-HCl (pH 7), 1 mM EDTA, 30% glycerol, and bromophenol blue) and 10 μl was loaded into the gel. Gels were run at 100 V for 1 h and 45 min in 1xTBE as the running buffer. Three microliters of RegeneTiss polyP standards p14 (20 mM), p60 (6.5 mM), and p130 (2.5 mM) were used. The gel was stained in fixing solution containing toluidine blue (25% methanol, 5% glycerol, and 0.05% toluidine blue) for 15 min and washed several times with destaining solution (fixing solution without toluidine blue) before being left overnight to fully destain.

### Western blotting

Cells were grown as indicated in figure legends and described in the “Bacterial strains and growth conditions” section. Cell pellets (3 OD_600_ equivalent of cells) frozen at −80°C were thawed on ice and resuspended in 100 μl sample buffer (800 μl sample buffer, 100 μl 1 M DTT, 100 μl 1.5 M Tris-HCl (pH 8.8)). Samples were boiled at 100°C for 10 min, then centrifuged at 13,000 rpm for 2 min and the supernatant was transferred to new tubes. Only ArnT and EptA membrane protein samples were prepared without boiling to prevent protein aggregation, typical for proteins containing transmembrane domains [84]. Cell pellets resuspended in 100 μl sample buffer were sonicated at power level 1 for 12 s, then centrifuged at 13,000 rpm for 2 min and the supernatant was transferred to new tubes. Protein samples were loaded on the indicated % of SDS-acrylamide gel. Proteins were transferred to PVDF membrane. Membranes were blocked for 20 min with shaking using TBST with 5% milk and washed 3 times for 10 min each with TBST after incubation with the primary and secondary antibodies. Blots were exposed to autoradiography film from Thomas Scientific. Conditions for antibody use can be found in S4 Table. Scanned images were opened in Photoshop. In most cases, small linear brightness and contrast adjustments were made to lighten the image background. Adjustments were applied evenly across the entire image shown.

### Liquid growth curves

Three biological replicates of each strain were cultured overnight in LB media. Overnights were diluted to 0.1 OD_600_ in LB and grown to mid-exponential phase (approximately 0.6 OD_600_). Cells (0.5 OD_600_ equivalent) were then washed twice with 1xPBS and resuspended in 50 μl of MOPS media (1xMOPS, 0.4% glucose, 0.1 mM K_2_HPO_4_) without amino acids. Twenty-five microliters of the cell suspension were transferred to 1 ml of MOPS without and with 0.05% or 0.5% amino acids (final OD approximately 0.1 OD_600_). Two hundred microliters of cells were pipetted in technical replicates of 3 and growth was monitored using the BioscreenC plate reader set at 37°C with continuous shaking. Optical density measurements were collected at a wavelength of 600 nm every 15 min, 5 s after shaking stopped, for 24 h. For the analysis, background OD values for each condition (0%, 0.05%, and 0.5% amino acids) were subtracted from each time point. See S2 Data for raw data.

### Growth curve analysis

Growth rate (OD_600_/min), lag time (min), and max OD_600_ measurements were calculated per well using GrowthRates 6.2 for Macintosh OS X [85] and Bare Bones Software text editor. GrowthRates was run using the stringent algorithm with the growth rate correlation coefficient set to r >0.99. For the analysis, the input file was in Standard Format and the program automatically compensated for the background OD at each time point. See S2 Data for raw data. Statistical analysis used two-way ANOVA with multiple comparisons, with correction for multiple hypothesis testing using Tukey’s test (GraphPad Prism Version 9.1.2).

### Reverse transcriptase quantitative PCR (RT-qPCR)

Five biological replicates of each strain were grown in LB or MOPS media for qPCR analysis. From each condition, 1 ml of cells were collected by centrifugation and immediately resuspended in 500 μl RNA*later* Solution for short-term storage at 4°C. Prior to RNA extraction. RNA*later* Solution was removed. RNA was extracted using the GeneJET RNA Purification Kit (Thermo Scientific) following manufacturer’s instructions and RNA integrity/concentration were evaluated by NanoDrop. Genomic DNA contamination was removed from RNA extracts by DNase treatment following the Invitrogen Ambion DNase I (RNase-free) protocol. DNase was removed using phenol-chloroform extraction as described previously [86]. Final RNA concentration was measured by NanoDrop. One microgram of RNA was reverse transcribed using SuperScript V VILO kit (Thermo Fisher) following manufacturer’s instructions (25°C for 10 min, 50°C for 10 min, and 85°C for 5 min). The cDNA was then diluted 1/10, aliquoted into 15 μl working solutions and stored at −80°C. We found that this dilution of cDNA was optimal to obtain Cq-values greater than 20 for our genes of interest. Quantitative PCRs were conducted in technical replicates of 3 and in a 10 μl final volume using iQ Sybr Green Supermix following manufacturer’s protocol under the following conditions: 95°C for 3 min and 39 cycles of 95°C for 15 s, 63°C for 30 s, and 72°C for 30 s. Melt curve analysis was performed at the end of each run and standard curves using serially diluted gDNA (extracted using One-4-All Genomic DNA MiniPreps Kit (BioBasic)) were performed to assess primer efficiency. All primers and primer efficiencies are reported in S3 Table. Gene expression was normalized to *yqfB*, used previously under polyP inducing conditions [69], and expression changes were calculated using the ΔΔC_T_ method using Bio-Rad CFX Maestro 2.3 version 5.3.002.1030 software. Statistical analysis used two-way ANOVA with multiple comparisons, with correction for multiple hypothesis testing using Tukey’s test (GraphPad Prism Version 9.1.2). See S3 Data for raw data.

### Polymyxin sensitivity

Indicated strains were streaked on LB or LB-kanamycin (plasmid carrying strains) plates and grown overnight at 37°C. The next day, a single colony from each strain was resuspended in 100 μl sterile water and serially diluted 10-fold in sterile water. Five microliters of each dilution were spotted onto the indicated plates that were prepared fresh on the day of use. Plates were allowed to dry prior to incubation at 37°C for 2 days (for MOPS plates) before imaging. Linear brightness and contrast adjustments made in Photoshop were made evenly across the entire image shown.

### Lipid analysis

Overnight cultures were diluted 1:50 in fresh LB medium. Cells were harvested at OD_600_ of 0.6 and washed with 1xPBS. Bacteria were resuspended in 1xMOPS medium supplemented with 0.4% glucose, 0.1 mM K2HPO4, and 2.5 μCi/ml ^32^Pi. Labeled cells were grown at 37°C and harvested after 6 h. Pellets were washed with 1xPBS. ^32^P-lipid A was extracted using mild acid hydrolysis as previously described [87]. The lipid A species were then resolved by TLC in a solvent system consisting of chloroform, pyridine, 88% formic acid, and water (50:50:16:5, vol/vol, respectively). The plates were exposed to a phosphor screen, and the radiolabeled lipids were visualized using an Amersham Typhoon phosphorimager system. Linear brightness and contrast adjustments were made in Photoshop to make clear the pEtN and L-Ara4N modifications. Linear adjustments were made evenly across the entire image shown.

### Databases

The mass spectrometry proteomics data developed for this study were deposited to the ProteomeXchange Consortium [88] via the PRIDE [89] partner repository with the data set identifier PXD043566.

## Supporting information

S1 Fig Wild-type *E*. *coli* accumulate polyP in MOPS minimal media while Δ*ppk* mutants do not. PolyP extraction gel from wild-type and Δ*ppk* mutant cultures used for mass spectrometry sample preparation. Overnight cultures were grown in LB media to mid-exponential phase and then shifted into MOPS minimal media for 3 h to induce starvation and polyP accumulation. PolyP extracts were run on a TBE-urea gel and stained with toluidine blue. The migration of a chain approximately 700 phosphate residues in length (p700) is indicated.
(TIF)

S2 Fig Arn expression is PPK-dependent during MOPS starvation. **(A)** Rescue of Arn expression following 3 h in MOPS media. Extracted protein samples were resolved using SDS-PAGE, transferred to PVDF, and detected using an anti-Flag antibody.
(TIF)

S3 Fig Molecular control of Arn and EptA protein expression by PPK. **(A)** Induction of ArnC-3Flag expression upon the switch from LB to MOPS media. The indicated strains were grown in LB media to mid-log phase and shifted to MOPS media for 3 h. Proteins were extracted and resolved via SDS-PAGE prior to transfer to a PVDF membrane. Tagged proteins were detected using an anti-Flag antibody. **(B)** Expression of PhoP between wild-type cells and Δ*ppk* mutants. The indicated strains were starved in MOPS media for 3 h prior to protein extraction, separation by SDS-PAGE, and detection with an antibody against PhoP. A background band (*) in Δ*phoP* mutants (controls used to validate the antibody) is PPK-regulated, which makes evaluation of changes to PhoP protein expression difficult. Regardless, regulation of PhoP by PPK appears to be minimal. **(C)** Expression of PhoQ-3Flag between wild-type cells and Δ*ppk* mutants. The indicated strains were starved in MOPS media for 3 h and proteins were analyzed as described in (B) using an antibody towards Flag. **(D)** Influence of magnesium (Mg^2+^) on ArnC-3Flag expression in MOPS media. Cells were grown to mid-exponential phase in LB and then shifted to MOPS minimal media in the absence or presence of 1 mM magnesium chloride or calcium chloride (control) for 3 h. Extracted protein samples were resolved using SDS-PAGE, transferred to PVDF, and detected using an anti-Flag antibody. Images shown are representative of results from ≥3 experiments. **(E)** Expression of RpoS between wild-type cells and Δ*ppk* mutants. The indicated strains were starved in MOPS media for 3 h prior to protein extraction, separation by SDS-PAGE, and detection with an antibody directed against RpoS. The Δ*rpoS* mutant strains serve to validate the antibody. **(F)** Expression of BasS-3Flag, BasR-3Flag, and ArnA-3Flag in LB supplemented with iron by wild-type and Δ*ppk* mutants. The indicated strains grown in LB or LB + iron (200 μm FeSO_4_) for 1.5 h prior to protein extraction, separation by SDS-PAGE, transfer to PVDF, and detection of tagged proteins with an anti-Flag antibody. **(G, H)** Influence of iron (G) and *phoB* mutation (H) on polyP accumulation. PolyP was extracted from the indicated strains grown in LB or LB + iron (200 μm FeSO_4_) for 1.5 h or in MOPS for 3 h following a shift from LB and analyzed on TBE-urea gels stained with toluidine blue. Note: the same BasR-tagged strains used for S2F were used for S2G polyP extraction, and the same ArnC-tagged strains used for 3I were used for the polyP extraction shown in S3H. Images shown are representative of results from ≥3 experiments, except for the polyP extractions in S3G and S3H, which are representative of 2 independent replicates.
(TIF)

S4 Fig Regulation of Arn protein expression and polymyxin resistance by PPK in the W3110 and WD101 (*basR*^*C*^) backgrounds. **(A)** PPK-dependent Arn-3Flag expression in W3110 and WD101 (*basR*^*C*^) strains. The indicated strains were grown in LB to mid log phase prior to shifting to MOPS for 3 h. Proteins were extracted and separated via SDS-PAGE prior to transfer to PVDF membrane and detection with anti-Flag antibody. **(B)** Time course of ArnC-3Flag expression following the shift from LB to MOPS media. Expression was analyzed for the indicated strains at the time points shown. Protein samples were resolved using a 12% SDS-PAGE gel, transferred to PVDF membrane, and proteins detected using an anti-Flag antibody. **(C)** Role of *ppk* in polymyxin resistance. Impact of *ppk* on the innate polymyxin resistance of W3110 and *basR*^*C*^ strains. The indicated strains were spotted in 10-fold serial dilutions on the indicated media and incubated at 37°C for 2 days prior to imaging. **(D)** Arn-dependence of polymyxin resistance in *basR*^*C*^ strains. Strains were diluted and grown as described in C. Images shown are representative of results from ≥3 experiments.
(TIF)

S5 Fig The role of PPK in the regulation of lipid A modification and polymyxin resistance. PolyP synthesized by PPK upon a switch from LB to MOPS media triggers BasS activation by autophosphorylation. Activated BasS then transphosphorylases BasR to induce downstream transcription of the *arnBCADTEF* operon and *EptA* gene. This results in increased level of Arn and EptA proteins, and up-regulation of the respective L-Ara4N and pEtN modifications. Dashed arrows indicate an additional step where the modified lipid A (a key structural component of the LPS) is transported to the outer membrane by the LPS transport system. This reduces the net negative charge of the outer membrane and results in polymyxin B (PMB) resistance. What is still unknown is whether polyP is acting directly in BasS activation and if PPK has a role independent of polyP synthesis.
(TIF)

S6 Fig Spent MOPS media from wild-type cultures does not induce Arn-3Flag expression of naïve cells. **(A, B)** Schematic (A) and western blotting (B) of media switch experiment. Cells were grown in LB media to mid-exponential phase and then shifted to MOPS media for 3 h to induce Arn expression (lanes 1 and 2). After 3 h in MOPS, spent media from wild-type cultures was centrifuged to remove cells and used for the media switch. The remainder of the culture was left to grow for another hour (lanes 5 and 6). For the media switch, cells exponentially growing in LB were pelleted, washed, and resuspended in the spent MOPS media from wild-type cultures. These cultures were left to grow for 1 h to test if the spent media contained the trigger needed to induce Arn expression (lanes 3 and 4). For western blotting, extracted protein samples were resolved using SDS-PAGE, transferred to PVDF, and detected using an anti-Flag antibody. Images shown are representative of results from ≥2 experiments.
(TIF)

S1 Table Mass spectrometry-identified proteins and differential expression analysis. (XLSX)

S2 Table Comparison of overlap between Varas and colleagues and Baijal and colleagues mass spectrometry data sets. (XLSX)

S3 Table Bacterial strains, plasmids, and qPCR primers used for this work. (XLSX)

S4 Table Reagents and antibody conditions used for this work. (XLSX)

S1 Data Raw data for volcano plot, all-or-none protein bubble plot, and GO term analysis presented in Fig 1B, 1C and 1E. (XLSX)

S2 Data Raw data for the growth curves and growth curve analysis presented in Fig 2A–2D. (XLSX)

S3 Data Underlying data for qPCR analysis presented in Fig 3G. (XLSX)

S1 Raw Images Uncropped and unadjusted western blot, ponceau S, polyP gel, lipid A profile, and spot test images. (PDF)

## Abbreviations

AGC: automatic gain control
CID: collision-induced dissociation
FDR: false discovery rate
GO: Gene Ontology
GSEA: Gene Set Enrichment Analysis
LPS: lipopolysaccharide
PPK: polyphosphate kinase
RT-qPCR: reverse transcriptase quantitative PCR
TCA: trichloroacetic acid
TEAB: tetraethylammonium bromide
TLC: thin-layer chromatography

## References

[1] Denoncourt A, Downey M. Model systems for studying polyphosphate biology: a focus on microorganisms. Curr Genet. 2021;67(3):331–46. Epub 2021/01/10. doi: 10.1007/s00294-020-01148-x . 33420907

[2] Desfougeres Y, Saiardi A, Azevedo C. Inorganic polyphosphate in mammals: where’s Wally? Biochem Soc Trans. 2020;48(1):95–101. Epub 2020/02/13. doi: 10.1042/BST20190328 . 32049314 PMC7054745

[3] Baijal K, Downey M. The promises of lysine polyphosphorylation as a regulatory modification in mammals are tempered by conceptual and technical challenges. Bioessays. 2021:e2100058. Epub 2021/05/18. doi: 10.1002/bies.202100058 . 33998006

[4] Rudat AK, Pokhrel A, Green TJ, Gray MJ. Mutations in Escherichia coli Polyphosphate Kinase That Lead to Dramatically Increased In Vivo Polyphosphate Levels. J Bacteriol. 2018;200(6). Epub 2018/01/10. doi: 10.1128/JB.00697-17 29311274 PMC5826030

[5] Gray MJ, Wholey WY, Wagner NO, Cremers CM, Mueller-Schickert A, Hock NT, et al. Polyphosphate is a primordial chaperone. Mol Cell. 2014;53(5):689–99. Epub 2014/02/25. doi: 10.1016/j.molcel.2014.01.012 24560923 PMC3996911

[6] Yoo NG, Dogra S, Meinen BA, Tse E, Haefliger J, Southworth DR, et al. Polyphosphate Stabilizes Protein Unfolding Intermediates as Soluble Amyloid-like Oligomers. J Mol Biol. 2018;430(21):4195–208. Epub 2018/08/22. doi: 10.1016/j.jmb.2018.08.016 30130556 PMC6186493

[7] Kuroda A, Tanaka S, Ikeda T, Kato J, Takiguchi N, Ohtake H. Inorganic polyphosphate kinase is required to stimulate protein degradation and for adaptation to amino acid starvation in Escherichia coli. Proc Natl Acad Sci U S A. 1999;96(25):14264–9. Epub 1999/12/10. doi: 10.1073/pnas.96.25.14264 10588694 PMC24425

[8] Yang ZX, Zhou YN, Yang Y, Jin DJ. Polyphosphate binds to the principal sigma factor of RNA polymerase during starvation response in Helicobacter pylori. Mol Microbiol. 2010;77(3):618–27. Epub 2010/06/18. doi: 10.1111/j.1365-2958.2010.07233.x 20553390 PMC2917625

[9] Shiba T, Tsutsumi K, Yano H, Ihara Y, Kameda A, Tanaka K, et al. Inorganic polyphosphate and the induction of rpoS expression. Proc Natl Acad Sci U S A. 1997;94(21):11210–5. Epub 1997/10/23. doi: 10.1073/pnas.94.21.11210 9326588 PMC23418

[10] McInerney P, Mizutani T, Shiba T. Inorganic polyphosphate interacts with ribosomes and promotes translation fidelity in vitro and in vivo. Mol Microbiol. 2006;60(2):438–47. Epub 2006/04/01. doi: 10.1111/j.1365-2958.2006.05103.x . 16573692

[11] Beaufay F, Amemiya HM, Guan J, Basalla J, Meinen BA, Chen Z, et al. Polyphosphate drives bacterial heterochromatin formation. Sci Adv. 2021;7(52):eabk0233. Epub 2021/12/23. doi: 10.1126/sciadv.abk0233 . 34936433 PMC10954037

[12] Kuroda A, Nomura K, Ohtomo R, Kato J, Ikeda T, Takiguchi N, et al. Role of inorganic polyphosphate in promoting ribosomal protein degradation by the Lon protease in E. coli. Science. 2001;293(5530):705–8. Epub 2001/07/28. doi: 10.1126/science.1061315 . 11474114

[13] Nomura K, Kato J, Takiguchi N, Ohtake H, Kuroda A. Effects of inorganic polyphosphate on the proteolytic and DNA-binding activities of Lon in Escherichia coli. J Biol Chem. 2004;279(33):34406–10. Epub 2004/06/10. doi: 10.1074/jbc.M404725200 . 15187082

[14] Gross MH, Konieczny I. Polyphosphate induces the proteolysis of ADP-bound fraction of initiator to inhibit DNA replication initiation upon stress in Escherichia coli. Nucleic Acids Res. 2020;48(10):5457–66. Epub 2020/04/14. doi: 10.1093/nar/gkaa217 32282902 PMC7261185

[15] Beaufay F, Quarles E, Franz A, Katamanin O, Wholey WY, Jakob U. Polyphosphate Functions In Vivo as an Iron Chelator and Fenton Reaction Inhibitor. mBio. 2020;11(4). Epub 2020/07/30. doi: 10.1128/mBio.01017-20 32723918 PMC7387796

[16] Dahl JU, Gray MJ, Bazopoulou D, Beaufay F, Lempart J, Koenigsknecht MJ, et al. The anti-inflammatory drug mesalamine targets bacterial polyphosphate accumulation. Nat Microbiol. 2017;2:16267. Epub 2017/01/24. doi: 10.1038/nmicrobiol.2016.267 28112760 PMC5514548

[17] Rashid MH, Rao NN, Kornberg A. Inorganic polyphosphate is required for motility of bacterial pathogens. J Bacteriol. 2000;182(1):225–7. Epub 1999/12/30. doi: 10.1128/JB.182.1.225-227.2000 10613886 PMC94263

[18] Roewe J, Stavrides G, Strueve M, Sharma A, Marini F, Mann A, et al. Bacterial polyphosphates interfere with the innate host defense to infection. Nat Commun. 2020;11(1):4035. Epub 2020/08/14. doi: 10.1038/s41467-020-17639-x 32788578 PMC7423913

[19] Tang-Fichaux M, Chagneau CV, Bossuet-Greif N, Nougayrede JP, Oswald E, Branchu P. The Polyphosphate Kinase of Escherichia coli Is Required for Full Production of the Genotoxin Colibactin. mSphere. 2020;5(6). Epub 2020/12/18. doi: 10.1128/mSphere.01195-20 33328353 PMC7771237

[20] Chen J, Su L, Wang X, Zhang T, Liu F, Chen H, et al. Polyphosphate Kinase Mediates Antibiotic Tolerance in Extraintestinal Pathogenic Escherichia coli PCN033. Front Microbiol. 2016;7:724. Epub 2016/06/01. doi: 10.3389/fmicb.2016.00724 27242742 PMC4871857

[21] Kuroda A, Kornberg A. Polyphosphate kinase as a nucleoside diphosphate kinase in Escherichia coli and Pseudomonas aeruginosa. Proc Natl Acad Sci U S A. 1997;94(2):439–42. Epub 1997/01/21. doi: 10.1073/pnas.94.2.439 9012801 PMC19530

[22] Du Y, Wang X, Han Z, Hua Y, Yan K, Zhang B, et al. Polyphosphate Kinase 1 Is a Pathogenesis Determinant in Enterohemorrhagic Escherichia coli O157:H7. Front Microbiol. 2021;12:762171. Epub 2021/11/16. doi: 10.3389/fmicb.2021.762171 34777317 PMC8578739

[23] Neville N, Roberge N, Ji X, Stephen P, Lu JL, Jia Z. A Dual-Specificity Inhibitor Targets Polyphosphate Kinase 1 and 2 Enzymes To Attenuate Virulence of Pseudomonas aeruginosa. mBio. 2021;12(3):e0059221. Epub 2021/06/16. doi: 10.1128/mBio.00592-21 34126765 PMC8262977

[24] Raetz CR, Reynolds CM, Trent MS, Bishop RE. Lipid A modification systems in gram-negative bacteria. Annu Rev Biochem. 2007;76:295–329. Epub 2007/03/17. doi: 10.1146/annurev.biochem.76.010307.145803 17362200 PMC2569861

[25] Rao NN, Liu S, Kornberg A. Inorganic polyphosphate in Escherichia coli: the phosphate regulon and the stringent response. J Bacteriol. 1998;180(8):2186–93. Epub 1998/04/29. doi: 10.1128/JB.180.8.2186-2193.1998 9555903 PMC107147

[26] Gray MJ. Inorganic Polyphosphate Accumulation in Escherichia coli Is Regulated by DksA but Not by (p)ppGpp. J Bacteriol. 2019;201(9). Epub 2019/02/13. doi: 10.1128/JB.00664-18 30745375 PMC6456864

[27] Varas M, Valdivieso C, Mauriaca C, Ortiz-Severin J, Paradela A, Poblete-Castro I, et al. Multi-level evaluation of Escherichia coli polyphosphate related mutants using global transcriptomic, proteomic and phenomic analyses. Biochim Biophys Acta Gen Subj. 2017;1861(4):871–83. Epub 2017/01/11. doi: 10.1016/j.bbagen.2017.01.007 . 28069396

[28] Ault-Riche D, Fraley CD, Tzeng CM, Kornberg A. Novel assay reveals multiple pathways regulating stress-induced accumulations of inorganic polyphosphate in Escherichia coli. J Bacteriol. 1998;180(7):1841–7. Epub 1998/04/16. doi: 10.1128/JB.180.7.1841-1847.1998 9537383 PMC107098

[29] Varas M, Valdivieso C, Mauriaca C, Ortiz-Severin J, Paradela A, Poblete-Castro I, et al. Datasets for transcriptomics, q-proteomics and phenotype microarrays of polyphosphate metabolism mutants from Escherichia coli. Data Brief. 2017;12:13–7. Epub 2017/04/05. doi: 10.1016/j.dib.2017.03.010 28373998 PMC5367803

[30] Ashburner M, Ball CA, Blake JA, Botstein D, Butler H, Cherry JM, et al. Gene ontology: tool for the unification of biology. The Gene Ontology Consortium. Nat Genet. 2000;25(1):25–9. Epub 2000/05/10. doi: 10.1038/75556 10802651 PMC3037419

[31] Subramanian A, Tamayo P, Mootha VK, Mukherjee S, Ebert BL, Gillette MA, et al. Gene set enrichment analysis: a knowledge-based approach for interpreting genome-wide expression profiles. Proc Natl Acad Sci U S A. 2005;102(43):15545–50. Epub 2005/10/04. doi: 10.1073/pnas.0506580102 16199517 PMC1239896

[32] Potrykus K, Cashel M. (p)ppGpp: still magical? Annu Rev Microbiol. 2008;62:35–51. Epub 2008/05/06. doi: 10.1146/annurev.micro.62.081307.162903 . 18454629

[33] Irving SE, Choudhury NR, Corrigan RM. The stringent response and physiological roles of (pp)pGpp in bacteria. Nat Rev Microbiol. 2021;19(4):256–71. Epub 2020/11/06. doi: 10.1038/s41579-020-00470-y . 33149273

[34] Parshin A, Shiver AL, Lee J, Ozerova M, Schneidman-Duhovny D, Gross CA, et al. DksA regulates RNA polymerase in Escherichia coli through a network of interactions in the secondary channel that includes Sequence Insertion 1. Proc Natl Acad Sci U S A. 2015;112(50):E6862–71. Epub 2015/11/26. doi: 10.1073/pnas.1521365112 26604313 PMC4687573

[35] Yang X, Ishiguro EE. Involvement of the N terminus of ribosomal protein L11 in regulation of the RelA protein of Escherichia coli. J Bacteriol. 2001;183(22):6532–7. Epub 2001/10/24. doi: 10.1128/JB.183.22.6532-6537.2001 11673421 PMC95482

[36] Kuroda A, Nomura K, Takiguchi N, Kato J, Ohtake H. Inorganic polyphosphate stimulates lon-mediated proteolysis of nucleoid proteins in Escherichia coli. Cell Mol Biol (Noisy-le-grand). 2006;52(4):23–9. Epub 2007/06/05. . 17543195

[37] Lee H, Hsu FF, Turk J, Groisman EA. The PmrA-regulated pmrC gene mediates phosphoethanolamine modification of lipid A and polymyxin resistance in Salmonella enterica. J Bacteriol. 2004;186(13):4124–33. Epub 2004/06/19. doi: 10.1128/JB.186.13.4124-4133.2004 15205413 PMC421605

[38] Breazeale SD, Ribeiro AA, Raetz CR. Origin of lipid A species modified with 4-amino-4-deoxy-L-arabinose in polymyxin-resistant mutants of Escherichia coli. An aminotransferase (ArnB) that generates UDP-4-deoxyl-L-arabinose. J Biol Chem. 2003;278(27):24731–9. Epub 2003/04/22. doi: 10.1074/jbc.M304043200 . 12704196

[39] Breazeale SD, Ribeiro AA, McClerren AL, Raetz CR. A formyltransferase required for polymyxin resistance in Escherichia coli and the modification of lipid A with 4-Amino-4-deoxy-L-arabinose. Identification and function oF UDP-4-deoxy-4-formamido-L-arabinose. J Biol Chem. 2005;280(14):14154–67. Epub 2005/02/08. doi: 10.1074/jbc.M414265200 . 15695810

[40] Gatzeva-Topalova PZ, May AP, Sousa MC. Structure and mechanism of ArnA: conformational change implies ordered dehydrogenase mechanism in key enzyme for polymyxin resistance. Structure. 2005;13(6):929–42. Epub 2005/06/09. doi: 10.1016/j.str.2005.03.018 15939024 PMC2997725

[41] Yan A, Guan Z, Raetz CR. An undecaprenyl phosphate-aminoarabinose flippase required for polymyxin resistance in Escherichia coli. J Biol Chem. 2007;282(49):36077–89. Epub 2007/10/12. doi: 10.1074/jbc.M706172200 17928292 PMC2613183

[42] Trent MS, Ribeiro AA, Lin S, Cotter RJ, Raetz CR. An inner membrane enzyme in Salmonella and Escherichia coli that transfers 4-amino-4-deoxy-L-arabinose to lipid A: induction on polymyxin-resistant mutants and role of a novel lipid-linked donor. J Biol Chem. 2001;276(46):43122–31. Epub 2001/09/06. doi: 10.1074/jbc.M106961200 . 11535604

[43] Kim SH, Jia W, Parreira VR, Bishop RE, Gyles CL. Phosphoethanolamine substitution in the lipid A of Escherichia coli O157: H7 and its association with PmrC. Microbiology (Reading). 2006;152 (Pt 3) 657–66. Epub 2006/03/04. doi: 10.1099/mic.0.28692-0 . 16514146

[44] Putker F, Bos MP, Tommassen J. Transport of lipopolysaccharide to the Gram-negative bacterial cell surface. FEMS Microbiol Rev. 2015;39(6):985–1002. Epub 2015/06/04. doi: 10.1093/femsre/fuv026 . 26038291

[45] Whitfield C, Trent MS. Biosynthesis and export of bacterial lipopolysaccharides. Annu Rev Biochem. 2014;83:99–128. Epub 2014/03/04. doi: 10.1146/annurev-biochem-060713-035600 . 24580642

[46] Velkov T, Deris ZZ, Huang JX, Azad MA, Butler M, Sivanesan S, et al. Surface changes and polymyxin interactions with a resistant strain of Klebsiella pneumoniae. Innate Immun. 2014;20(4):350–63. Epub 2013/07/28. doi: 10.1177/1753425913493337 23887184 PMC4242413

[47] Hagiwara D, Yamashino T, Mizuno T. A Genome-wide view of the Escherichia coli BasS-BasR two-component system implicated in iron-responses. Biosci Biotechnol Biochem. 2004;68(8):1758–67. Epub 2004/08/24. doi: 10.1271/bbb.68.1758 . 15322361

[48] Ogasawara H, Shinohara S, Yamamoto K, Ishihama A. Novel regulation targets of the metal-response BasS-BasR two-component system of Escherichia coli. Microbiology (Reading). 2012;158(Pt 6):1482–92. Epub 2012/03/24. doi: 10.1099/mic.0.057745-0 . 22442305

[49] Lee LJ, Barrett JA, Poole RK. Genome-wide transcriptional response of chemostat-cultured Escherichia coli to zinc. J Bacteriol. 2005;187(3):1124–34. Epub 2005/01/22. doi: 10.1128/JB.187.3.1124-1134.2005 15659689 PMC545701

[50] Wosten MM, Kox LF, Chamnongpol S, Soncini FC, Groisman EA. A signal transduction system that responds to extracellular iron. Cell. 2000;103(1):113–25. Epub 2000/10/29. doi: 10.1016/s0092-8674(00)00092-1 . 11051552

[51] Rubin EJ, Herrera CM, Crofts AA, Trent MS. PmrD is required for modifications to escherichia coli endotoxin that promote antimicrobial resistance. Antimicrob Agents Chemother. 2015;59(4):2051–61. Epub 2015/01/22. doi: 10.1128/AAC.05052-14 25605366 PMC4356761

[52] Garcia Vescovi E, Soncini FC, Groisman EA. Mg2+ as an extracellular signal: environmental regulation of Salmonella virulence. Cell. 1996;84(1):165–74. Epub 1996/01/12. doi: 10.1016/s0092-8674(00)81003-x . 8548821

[53] Conway T, Creecy JP, Maddox SM, Grissom JE, Conkle TL, Shadid TM, et al. Unprecedented high-resolution view of bacterial operon architecture revealed by RNA sequencing. mBio. 2014;5(4):e01442–14. Epub 2014/07/10. doi: 10.1128/mBio.01442-14 25006232 PMC4161252

[54] Liu X, Xu J, Zhu J, Du P, Sun A. Combined Transcriptome and Proteome Analysis of RpoS Regulon Reveals Its Role in Spoilage Potential of Pseudomonas fluorescens. Front Microbiol. 2019;10:94. Epub 2019/02/23. doi: 10.3389/fmicb.2019.00094 30787912 PMC6372562

[55] Wong GT, Bonocora RP, Schep AN, Beeler SM, Lee Fong AJ, Shull LM, et al. Genome-Wide Transcriptional Response to Varying RpoS Levels in Escherichia coli K-12. J Bacteriol. 2017;199(7). Epub 2017/01/25. doi: 10.1128/JB.00755-16 28115545 PMC5350281

[56] Herrera CM, Hankins JV, Trent MS. Activation of PmrA inhibits LpxT-dependent phosphorylation of lipid A promoting resistance to antimicrobial peptides. Mol Microbiol. 2010;76(6):1444–60. Epub 2010/04/14. doi: 10.1111/j.1365-2958.2010.07150.x 20384697 PMC2904496

[57] Wang J, Ma W, Wang Z, Li Y, Wang X. Construction and characterization of an Escherichia coli mutant producing Kdo(2)-lipid A. Mar Drugs. 2014;12(3):1495–511. Epub 2014/03/19. doi: 10.3390/md12031495 24633251 PMC3967223

[58] Carty SM, Sreekumar KR, Raetz CR. Effect of cold shock on lipid A biosynthesis in Escherichia coli. Induction At 12 degrees C of an acyltransferase specific for palmitoleoyl-acyl carrier protein. J Biol Chem. 1999;274(14):9677–85. Epub 1999/03/27. doi: 10.1074/jbc.274.14.9677 . 10092655

[59] Moffatt JH, Harper M, Boyce JD. Mechanisms of Polymyxin Resistance. Adv Exp Med Biol. 2019;1145:55–71. Epub 2019/08/01. doi: 10.1007/978-3-030-16373-0_5 . 31364071

[60] Daugelavicius R, Bakiene E, Bamford DH. Stages of polymyxin B interaction with the Escherichia coli cell envelope. Antimicrob Agents Chemother. 2000;44(11):2969–78. Epub 2000/10/19. doi: 10.1128/AAC.44.11.2969-2978.2000 11036008 PMC101588

[61] Manioglu S, Modaresi SM, Ritzmann N, Thoma J, Overall SA, Harms A, et al. Antibiotic polymyxin arranges lipopolysaccharide into crystalline structures to solidify the bacterial membrane. Nat Commun. 2022;13(1):6195. Epub 2022/10/22. doi: 10.1038/s41467-022-33838-0 36271003 PMC9587031

[62] Ayoub Moubareck C. Polymyxins and Bacterial Membranes: A Review of Antibacterial Activity and Mechanisms of Resistance. Membranes (Basel). 2020;10(8). Epub 2020/08/14. doi: 10.3390/membranes10080181 32784516 PMC7463838

[63] Trent MS, Ribeiro AA, Doerrler WT, Lin S, Cotter RJ, Raetz CR. Accumulation of a polyisoprene-linked amino sugar in polymyxin-resistant Salmonella typhimurium and Escherichia coli: structural characterization and transfer to lipid A in the periplasm. J Biol Chem. 2001;276(46):43132–44. Epub 2001/09/06. doi: 10.1074/jbc.M106962200 . 11535605

[64] Nguyen D, Joshi-Datar A, Lepine F, Bauerle E, Olakanmi O, Beer K, et al. Active starvation responses mediate antibiotic tolerance in biofilms and nutrient-limited bacteria. Science. 2011;334(6058):982–6. Epub 2011/11/19. doi: 10.1126/science.1211037 22096200 PMC4046891

[65] Ito A, Taniuchi A, May T, Kawata K, Okabe S. Increased antibiotic resistance of Escherichia coli in mature biofilms. Appl Environ Microbiol. 2009;75(12):4093–100. Epub 2009/04/21. doi: 10.1128/AEM.02949-08 19376922 PMC2698376

[66] Schembri MA, Kjaergaard K, Klemm P. Global gene expression in Escherichia coli biofilms. Mol Microbiol. 2003;48(1):253–67. Epub 2003/03/27. doi: 10.1046/j.1365-2958.2003.03432.x . 12657059

[67] Gray MJ. Interactions between DksA and Stress-Responsive Alternative Sigma Factors Control Inorganic Polyphosphate Accumulation in Escherichia coli. J Bacteriol. 2020;202(14). Epub 2020/04/29. doi: 10.1128/JB.00133-20 32341074 PMC7317045

[68] Downey M. A Stringent Analysis of Polyphosphate Dynamics in Escherichia coli. J Bacteriol. 2019;201(9). Epub 2019/02/21. doi: 10.1128/JB.00070-19 30782636 PMC6456866

[69] Bowlin MQ, Long AR, Huffines JT, Gray MJ. The role of nitrogen-responsive regulators in controlling inorganic polyphosphate synthesis in Escherichia coli. Microbiology (Reading). 2022;168(4). Epub 2022/04/29. doi: 10.1099/mic.0.001185 35482529 PMC10233264

[70] Kato A, Latifi T, Groisman EA. Closing the loop: the PmrA/PmrB two-component system negatively controls expression of its posttranscriptional activator PmrD. Proc Natl Acad Sci U S A. 2003;100(8):4706–11. Epub 2003/04/05. doi: 10.1073/pnas.0836837100 12676988 PMC153620

[71] Datsenko KA, Wanner BL. One-step inactivation of chromosomal genes in Escherichia coli K-12 using PCR products. Proc Natl Acad Sci U S A. 2000;97(12):6640–5. Epub 2000/06/01. doi: 10.1073/pnas.120163297 10829079 PMC18686

[72] Datta S, Costantino N, Court DL. A set of recombineering plasmids for gram-negative bacteria. Gene. 2006;379:109–15. Epub 2006/06/06. doi: 10.1016/j.gene.2006.04.018 . 16750601

[73] Uzzau S, Figueroa-Bossi N, Rubino S, Bossi L. Epitope tagging of chromosomal genes in Salmonella. Proc Natl Acad Sci U S A. 2001;98(26):15264–9. Epub 2001/12/14. doi: 10.1073/pnas.261348198 11742086 PMC65018

[74] Cherepanov PP, Wackernagel W. Gene disruption in Escherichia coli: TcR and KmR cassettes with the option of Flp-catalyzed excision of the antibiotic-resistance determinant. Gene. 1995;158(1):9–14. Epub 1995/05/26. doi: 10.1016/0378-1119(95)00193-a 7789817

[75] Sharan SK, Thomason LC, Kuznetsov SG, Court DL. Recombineering: a homologous recombination-based method of genetic engineering. Nat Protoc. 2009;4(2):206–23. Epub 2009/01/31. doi: 10.1038/nprot.2008.227 19180090 PMC2790811

[76] Deutsch EW, Mendoza L, Shteynberg DD, Hoopmann MR, Sun Z, Eng JK, et al. Trans-Proteomic Pipeline: Robust Mass Spectrometry-Based Proteomics Data Analysis Suite. J Proteome Res. 2023;22(2):615–24. Epub 2023/01/18. doi: 10.1021/acs.jproteome.2c00624 36648445 PMC10166710

[77] Chambers MC, Maclean B, Burke R, Amodei D, Ruderman DL, Neumann S, et al. A cross-platform toolkit for mass spectrometry and proteomics. Nat Biotechnol. 2012;30(10):918–20. Epub 2012/10/12. doi: 10.1038/nbt.2377 23051804 PMC3471674

[78] Eng JK, Jahan TA, Hoopmann MR. Comet: an open-source MS/MS sequence database search tool. Proteomics. 2013;13(1):22–4. Epub 2012/11/14. doi: 10.1002/pmic.201200439 . 23148064

[79] UniProt C. UniProt: the Universal Protein Knowledgebase in 2023. Nucleic Acids Res. 2023;51(D1):D523–D31. Epub 2022/11/22. doi: 10.1093/nar/gkac1052 36408920 PMC9825514

[80] Nesvizhskii AI, Keller A, Kolker E, Aebersold R. A statistical model for identifying proteins by tandem mass spectrometry. Anal Chem. 2003;75(17):4646–58. Epub 2003/11/25. doi: 10.1021/ac0341261 . 14632076

[81] Benjamini Y, Hochberg Y. Controlling the false discovery rate: a practical and powerful approach to multiple testing. J R Stat Soc Series B. 1995;57(1):289–300.

[82] Bauer S, Grossmann S, Vingron M, Robinson PN. Ontologizer 2.0—a multifunctional tool for GO term enrichment analysis and data exploration. Bioinformatics. 2008;24(14):1650–1. Epub 2008/05/31. doi: 10.1093/bioinformatics/btn250 . 18511468

[83] Bondy-Chorney E, Abramchuk I, Nasser R, Holinier C, Denoncourt A, Baijal K, et al. A Broad Response to Intracellular Long-Chain Polyphosphate in Human Cells. Cell Rep. 2020;33(4):108318. Epub 2020/10/29. doi: 10.1016/j.celrep.2020.108318 . 33113373

[84] Karginov A, Agaphonov M. A simple enrichment procedure improves detection of membrane proteins by immunoblotting. Biotechniques. 2016;61(5):260–1. Epub 2016/11/15. doi: 10.2144/000114474 . 27839511

[85] Hall BG, Acar H, Nandipati A, Barlow M. Growth rates made easy. Mol Biol Evol. 2014;31(1):232–8. Epub 2013/10/31. doi: 10.1093/molbev/mst187 . 24170494

[86] McCarthy L, Abramchuk I, Wafy G, Denoncourt A, Lavallee-Adam M, Downey M. Ddp1 Cooperates with Ppx1 to Counter a Stress Response Initiated by Nonvacuolar Polyphosphate. mBio. 2022:e0039022. Epub 2022/07/22. doi: 10.1128/mbio.00390-22 . 35862758 PMC9426566

[87] Herrera CM, Voss BJ, Trent MS. Homeoviscous Adaptation of the Acinetobacter baumannii Outer Membrane: Alteration of Lipooligosaccharide Structure during Cold Stress. mBio. 2021;12(4):e0129521. Epub 2021/08/25. doi: 10.1128/mBio.01295-21 34425709 PMC8406137

[88] Deutsch EW, Csordas A, Sun Z, Jarnuczak A, Perez-Riverol Y, Ternent T, et al. The ProteomeXchange consortium in 2017: supporting the cultural change in proteomics public data deposition. Nucleic Acids Res. 2017;45(D1):D1100–D6. Epub 2016/12/08. doi: 10.1093/nar/gkw936 27924013 PMC5210636

[89] Perez-Riverol Y, Csordas A, Bai J, Bernal-Llinares M, Hewapathirana S, Kundu DJ, et al. The PRIDE database and related tools and resources in 2019: improving support for quantification data. Nucleic Acids Res. 2019;47(D1):D442–D50. Epub 2018/11/06. doi: 10.1093/nar/gky1106 30395289 PMC6323896

