# Supplementary figures and images for: Polyphosphate kinase regulates LPS structure and polymyxin resistance during starvation in E. coli
Source: PLoS Biol. 2024 Mar 13;22(3):e3002558. doi: 10.1371/journal.pbio.3002558 (PMC10962826; doi:10.1371/journal.pbio.3002558)

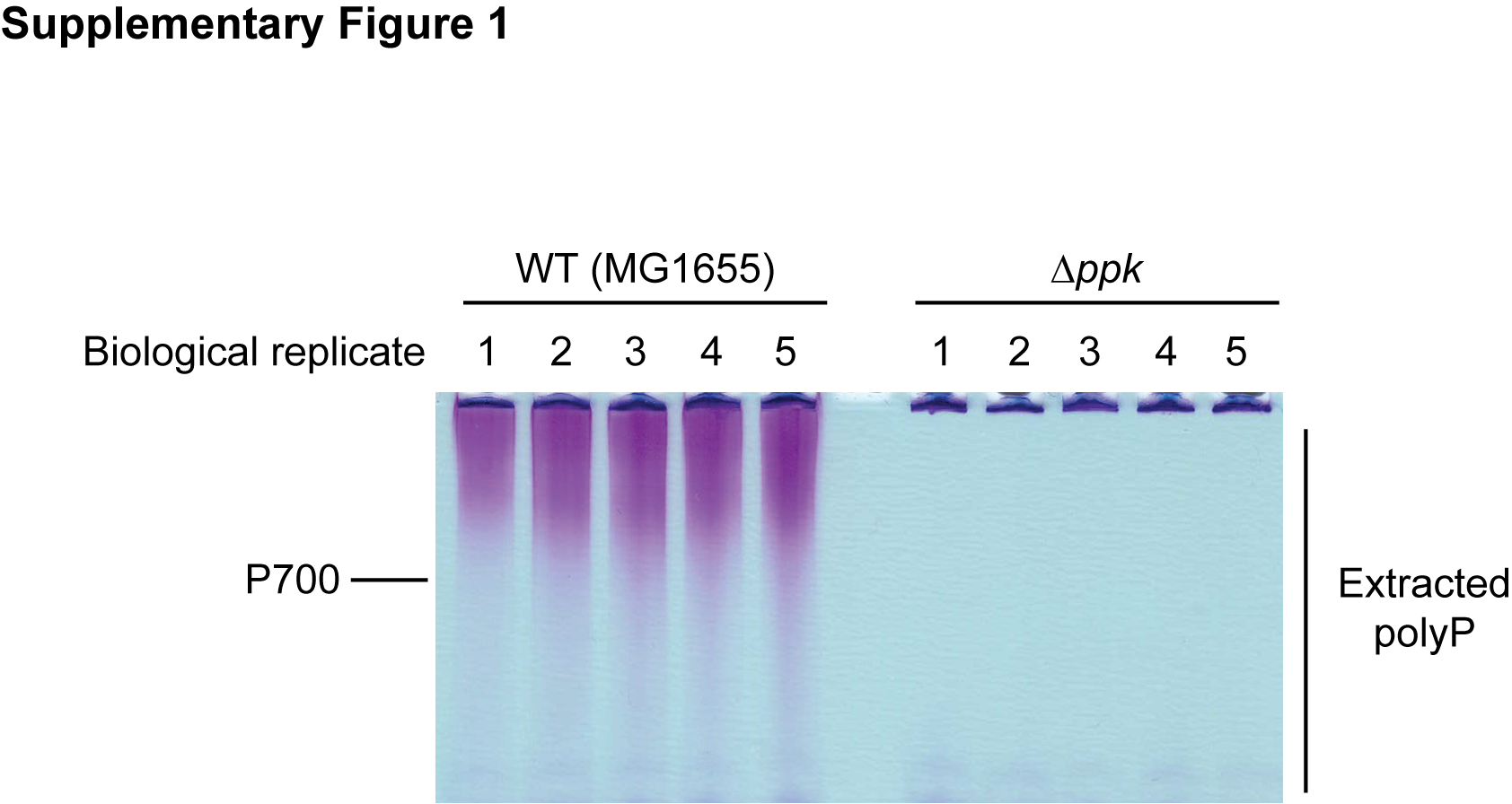

Supplement: S1 Fig — PolyP extraction gel from wild-type and Δppk mutant cultures used for mass spectrometry sample preparation. Overnight cultures were grown in LB media to mid-exponential phase and then shifted into MOPS minimal media for 3 h to induce starvation and polyP accumulation. PolyP extracts were run on a TBE-urea gel and stained with toluidine blue. The migration of a chain approximately 700 phosphate residues in length (p700) is indicated. (TIF) [file pbio.3002558.s001.tif]

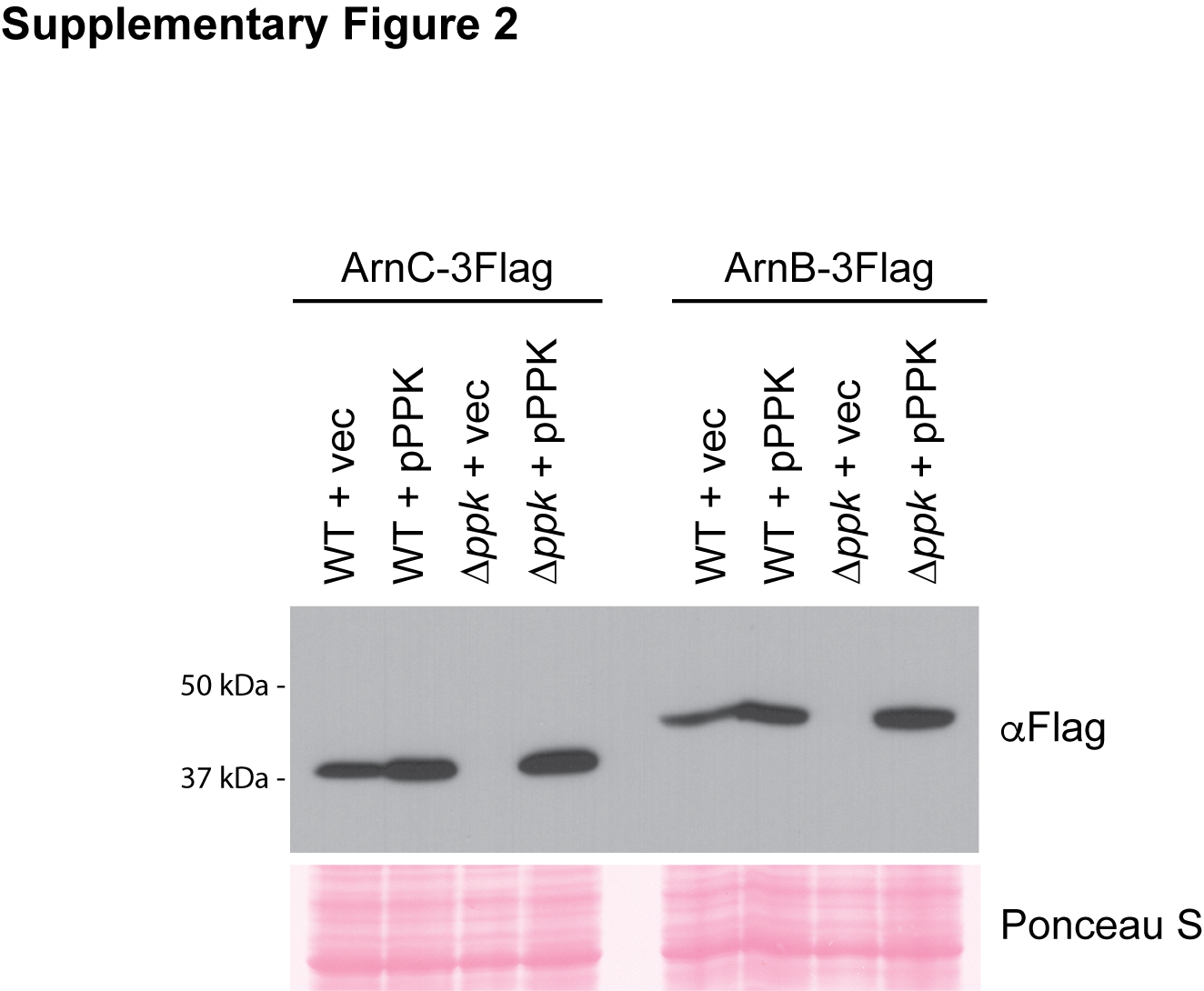

Supplement: S2 Fig — (A) Rescue of Arn expression following 3 h in MOPS media. Extracted protein samples were resolved using SDS-PAGE, transferred to PVDF, and detected using an anti-Flag antibody. (TIF) [file pbio.3002558.s002.tif]

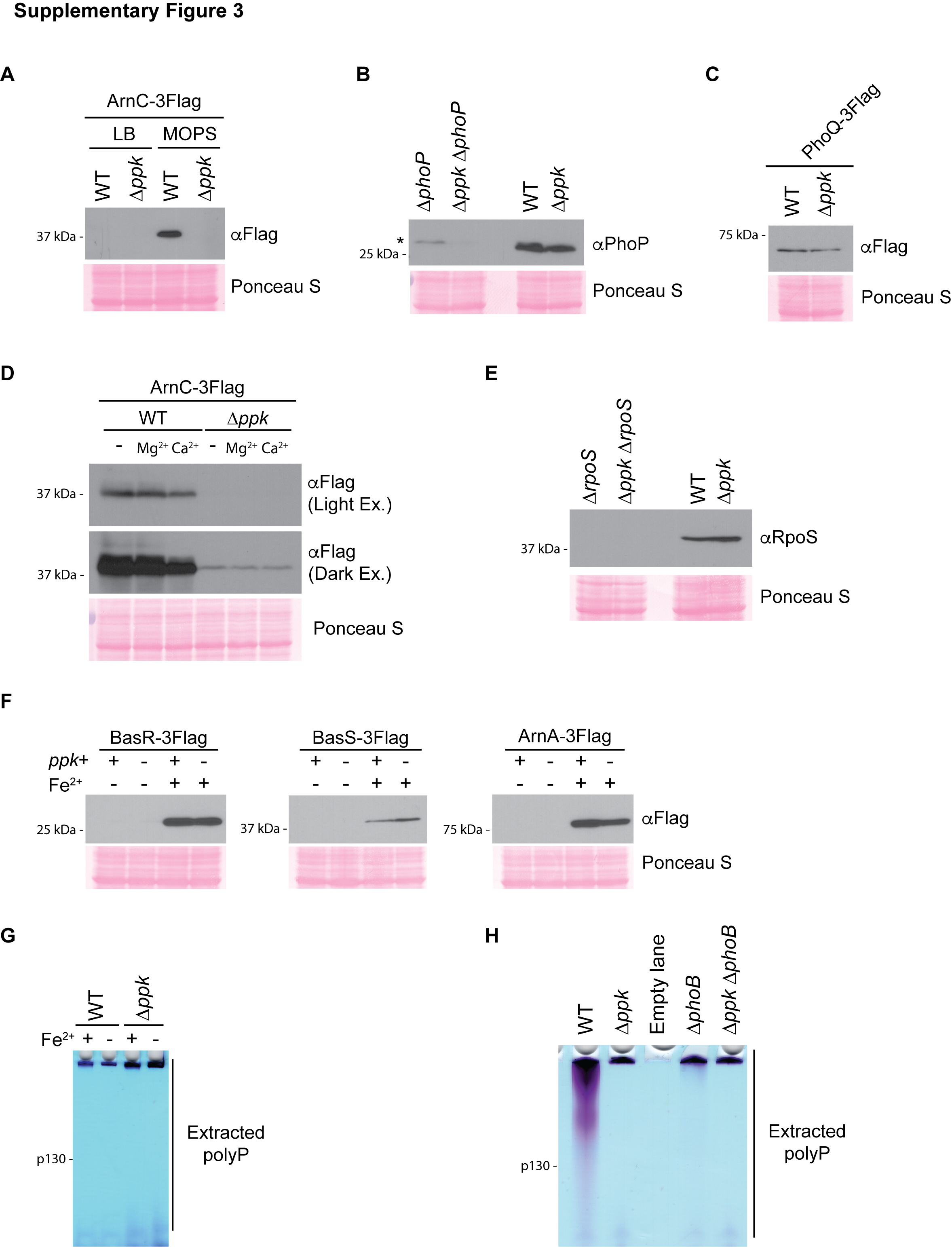

Supplement: S3 Fig — (A) Induction of ArnC-3Flag expression upon the switch from LB to MOPS media. The indicated strains were grown in LB media to mid-log phase and shifted to MOPS media for 3 h. Proteins were extracted and resolved via SDS-PAGE prior to transfer to a PVDF membrane. Tagged proteins were detected using an anti-Flag antibody. (B) Expression of PhoP between wild-type cells and Δppk mutants. The indicated strains were starved in MOPS media for 3 h prior to protein extraction, separation by SDS-PAGE, and detection with an antibody against PhoP. A background band (*) in ΔphoP mutants (controls used to validate the antibody) is PPK-regulated, which makes evaluation of changes to PhoP protein expression difficult. Regardless, regulation of PhoP by PPK appears to be minimal. (C) Expression of PhoQ-3Flag between wild-type cells and Δppk mutants. The indicated strains were starved in MOPS media for 3 h and proteins were analyzed as described in (B) using an antibody towards Flag. (D) Influence of magnesium (Mg2+) on ArnC-3Flag expression in MOPS media. Cells were grown to mid-exponential phase in LB and then shifted to MOPS minimal media in the absence or presence of 1 mM magnesium chloride or calcium chloride (control) for 3 h. Extracted protein samples were resolved using SDS-PAGE, transferred to PVDF, and detected using an anti-Flag antibody. Images shown are representative of results from ≥3 experiments. (E) Expression of RpoS between wild-type cells and Δppk mutants. The indicated strains were starved in MOPS media for 3 h prior to protein extraction, separation by SDS-PAGE, and detection with an antibody directed against RpoS. The ΔrpoS mutant strains serve to validate the antibody. (F) Expression of BasS-3Flag, BasR-3Flag, and ArnA-3Flag in LB supplemented with iron by wild-type and Δppk mutants. The indicated strains grown in LB or LB + iron (200 μm FeSO4) for 1.5 h prior to protein extraction, separation by SDS-PAGE, transfer to PVDF, and detection of tagged proteins with [file pbio.3002558.s003.tif]

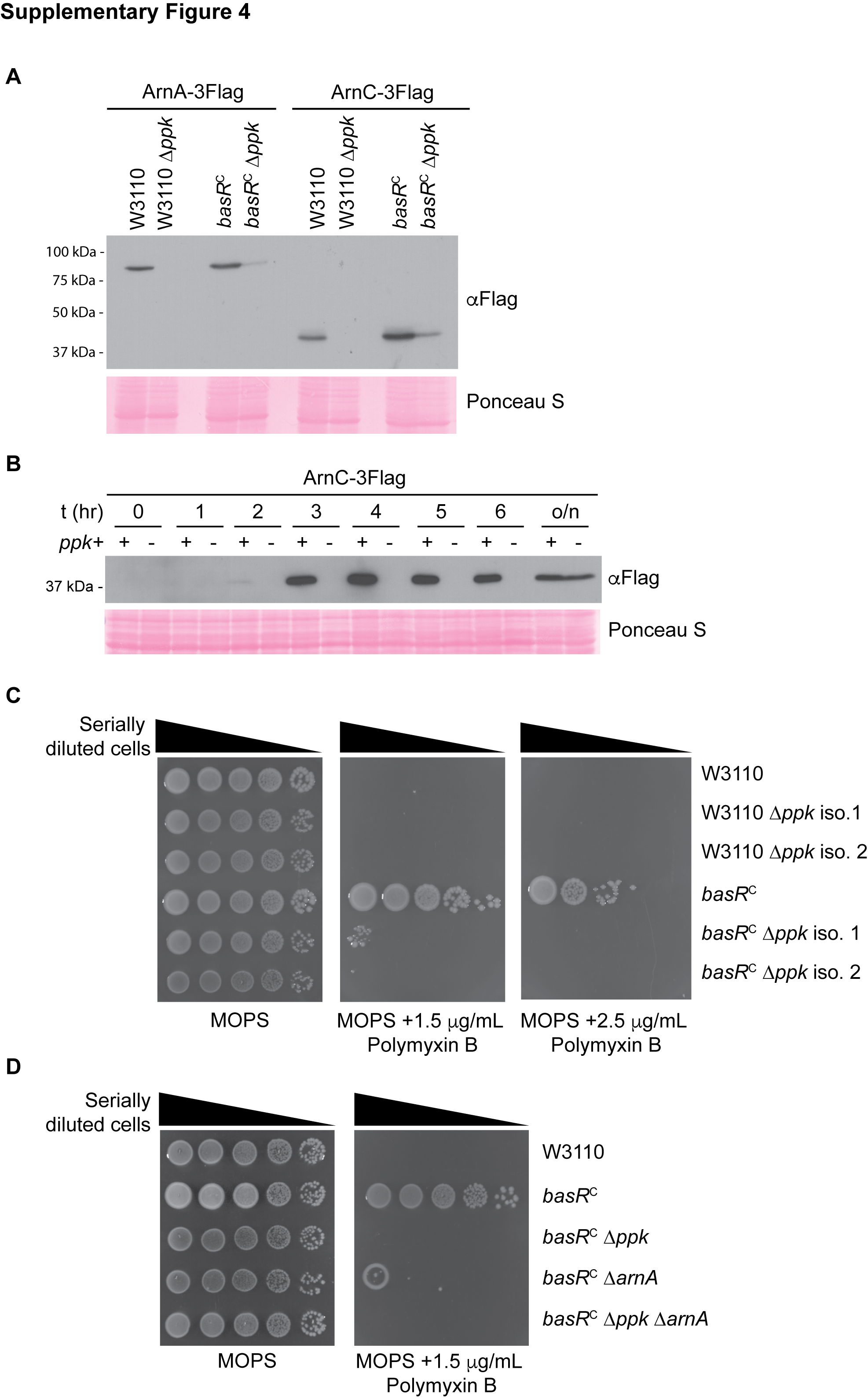

Supplement: S4 Fig — (A) PPK-dependent Arn-3Flag expression in W3110 and WD101 (basRC) strains. The indicated strains were grown in LB to mid log phase prior to shifting to MOPS for 3 h. Proteins were extracted and separated via SDS-PAGE prior to transfer to PVDF membrane and detection with anti-Flag antibody. (B) Time course of ArnC-3Flag expression following the shift from LB to MOPS media. Expression was analyzed for the indicated strains at the time points shown. Protein samples were resolved using a 12% SDS-PAGE gel, transferred to PVDF membrane, and proteins detected using an anti-Flag antibody. (C) Role of ppk in polymyxin resistance. Impact of ppk on the innate polymyxin resistance of W3110 and basRC strains. The indicated strains were spotted in 10-fold serial dilutions on the indicated media and incubated at 37°C for 2 days prior to imaging. (D) Arn-dependence of polymyxin resistance in basRC strains. Strains were diluted and grown as described in C. Images shown are representative of results from ≥3 experiments. (TIF) [file pbio.3002558.s004.tif]

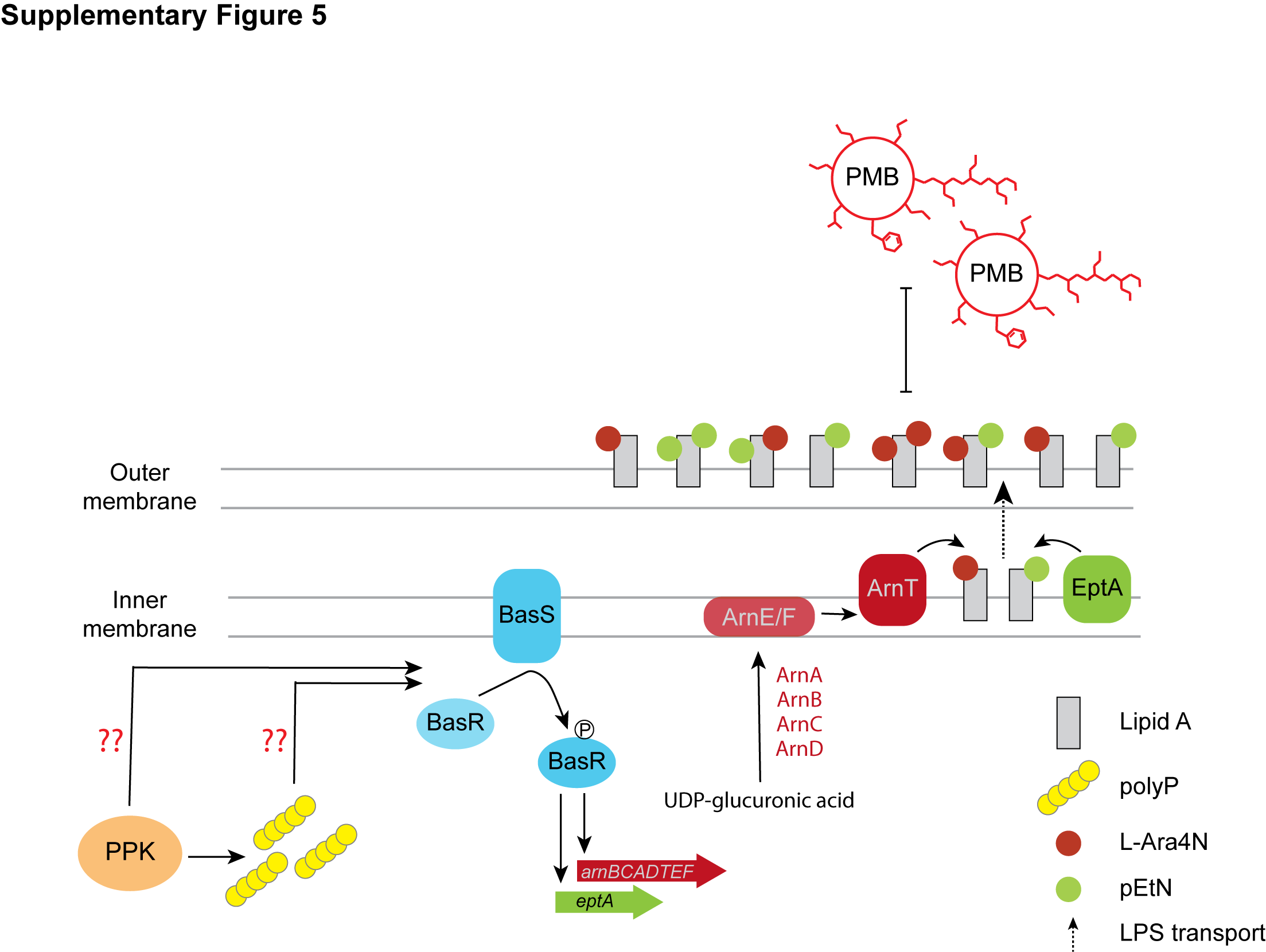

Supplement: S5 Fig — PolyP synthesized by PPK upon a switch from LB to MOPS media triggers BasS activation by autophosphorylation. Activated BasS then transphosphorylases BasR to induce downstream transcription of the arnBCADTEF operon and EptA gene. This results in increased level of Arn and EptA proteins, and up-regulation of the respective L-Ara4N and pEtN modifications. Dashed arrows indicate an additional step where the modified lipid A (a key structural component of the LPS) is transported to the outer membrane by the LPS transport system. This reduces the net negative charge of the outer membrane and results in polymyxin B (PMB) resistance. What is still unknown is whether polyP is acting directly in BasS activation and if PPK has a role independent of polyP synthesis. (TIF) [file pbio.3002558.s005.tif]

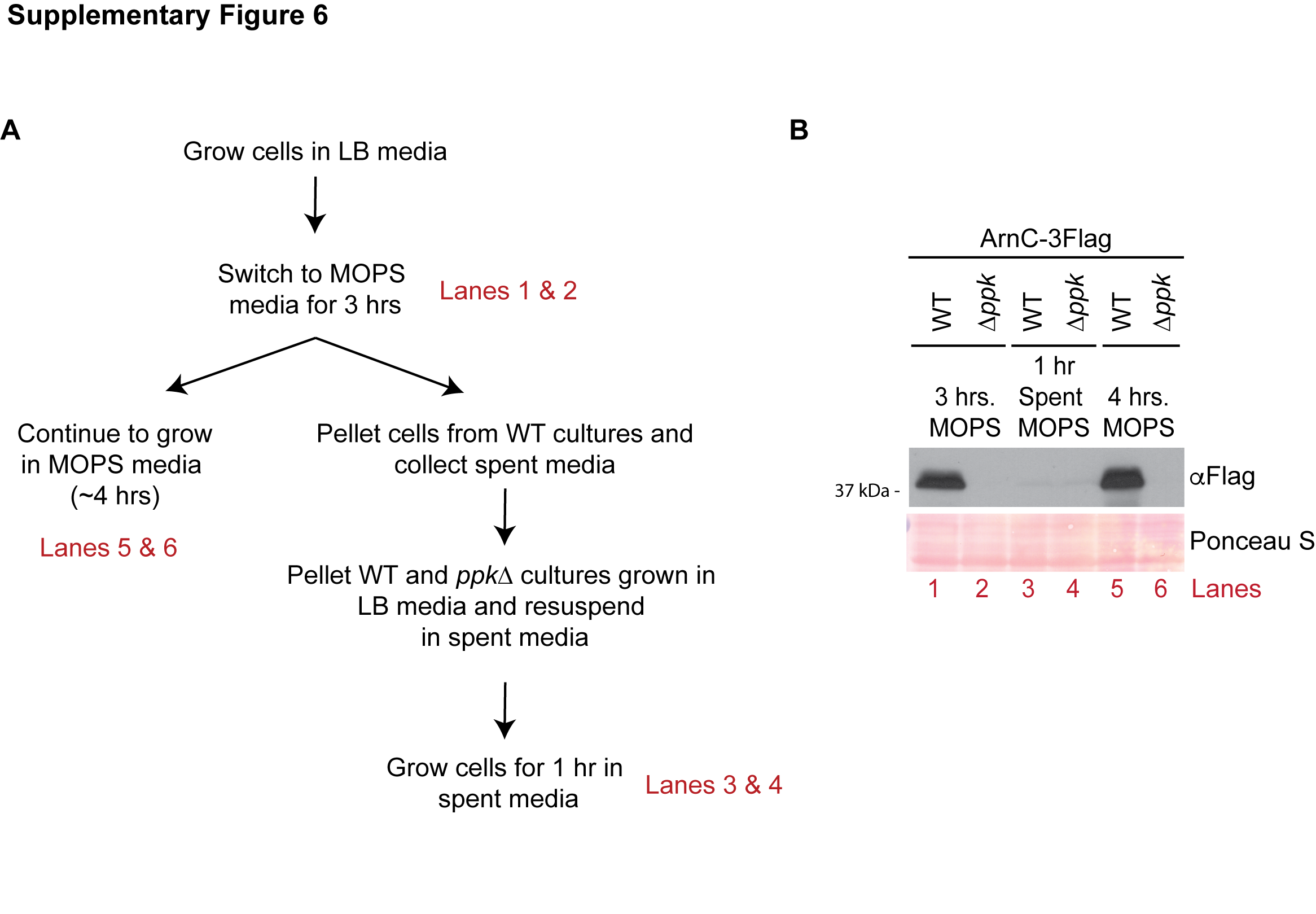

Supplement: S6 Fig — (A, B) Schematic (A) and western blotting (B) of media switch experiment. Cells were grown in LB media to mid-exponential phase and then shifted to MOPS media for 3 h to induce Arn expression (lanes 1 and 2). After 3 h in MOPS, spent media from wild-type cultures was centrifuged to remove cells and used for the media switch. The remainder of the culture was left to grow for another hour (lanes 5 and 6). For the media switch, cells exponentially growing in LB were pelleted, washed, and resuspended in the spent MOPS media from wild-type cultures. These cultures were left to grow for 1 h to test if the spent media contained the trigger needed to induce Arn expression (lanes 3 and 4). For western blotting, extracted protein samples were resolved using SDS-PAGE, transferred to PVDF, and detected using an anti-Flag antibody. Images shown are representative of results from ≥2 experiments. (TIF) [file pbio.3002558.s006.tif]
