# Supplementary material for: Polyphosphate kinase regulates LPS structure and polymyxin resistance during starvation in E. coli
Source: PLoS Biol. 2024 Mar 13;22(3):e3002558. doi: 10.1371/journal.pbio.3002558 (PMC10962826; doi:10.1371/journal.pbio.3002558)

Raw data for Figure 1.

Used for ArnC, ArnB and OtsA (Fig. 1D)

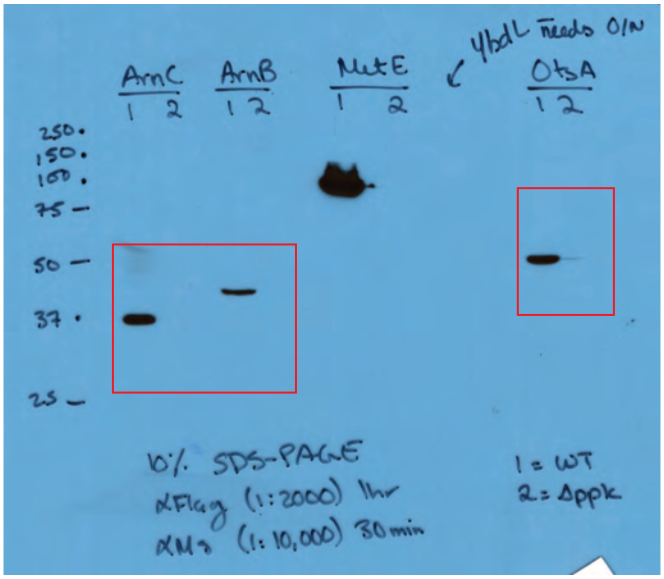

Used for MetE (lighter exposure of film on the left) (Fig. 1D)

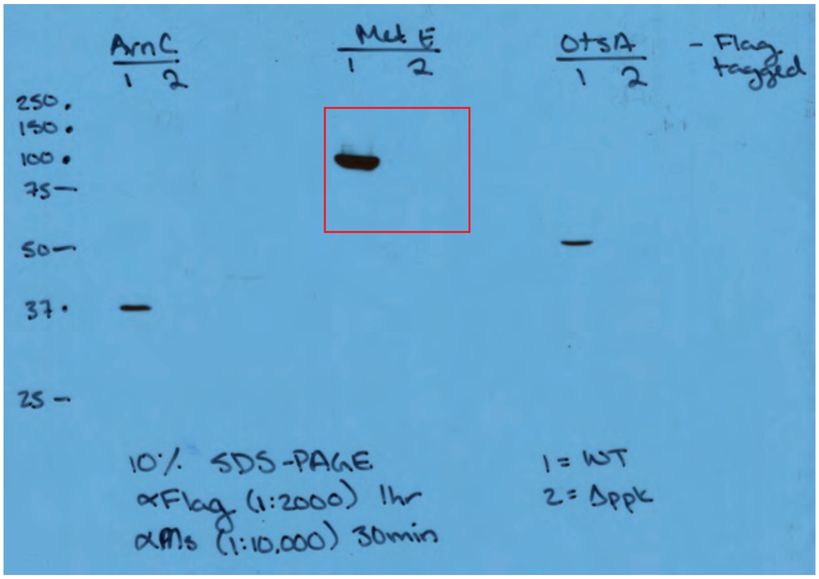

Corresponding ponceau for films above.

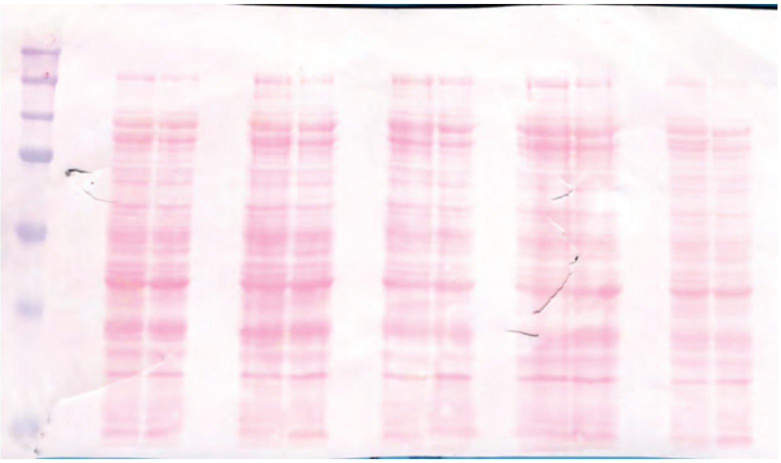

Used for YbdL (Fig. 1D)

YbdL-3xFlag

WT *ppk*

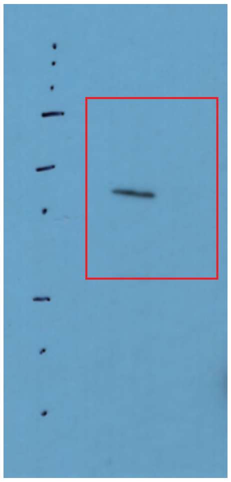

Ponceau corresponding to YbdL blot

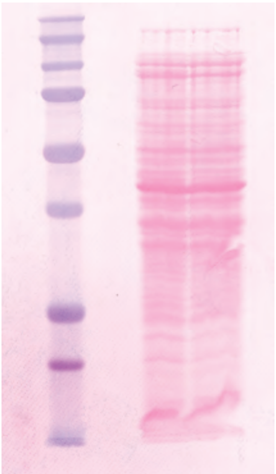

Used for YeaG (Fig. 1D)

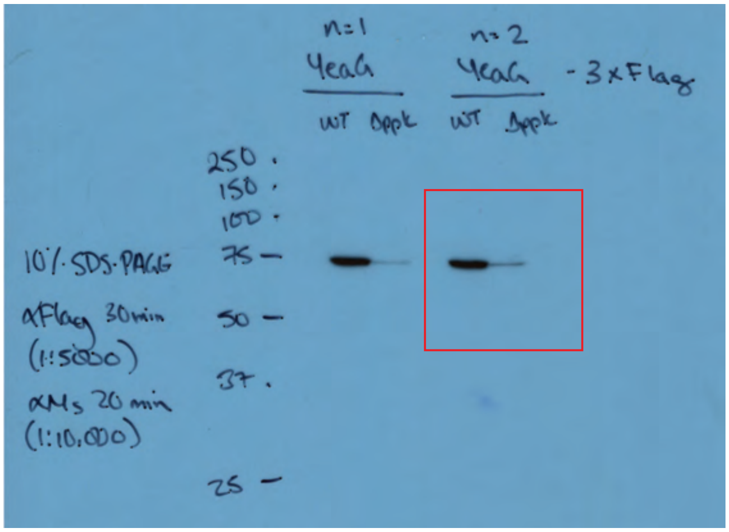

Ponceau corresponding to YeaG blot

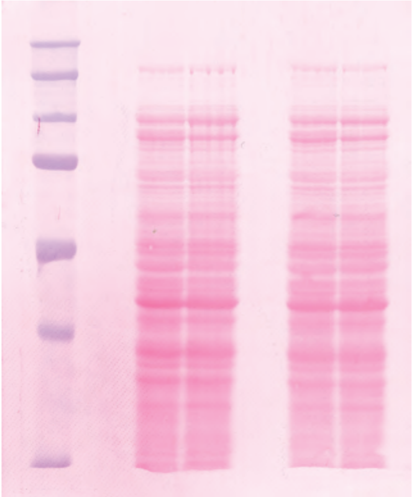

Raw data for figure 2 western blots.

Exposure used for MetE (Fig. 2E)

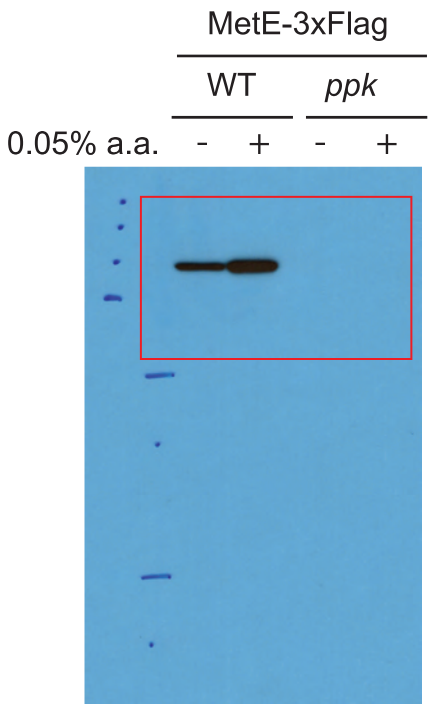

Exposure used for OtsA and YeaG (Fig. 2E)

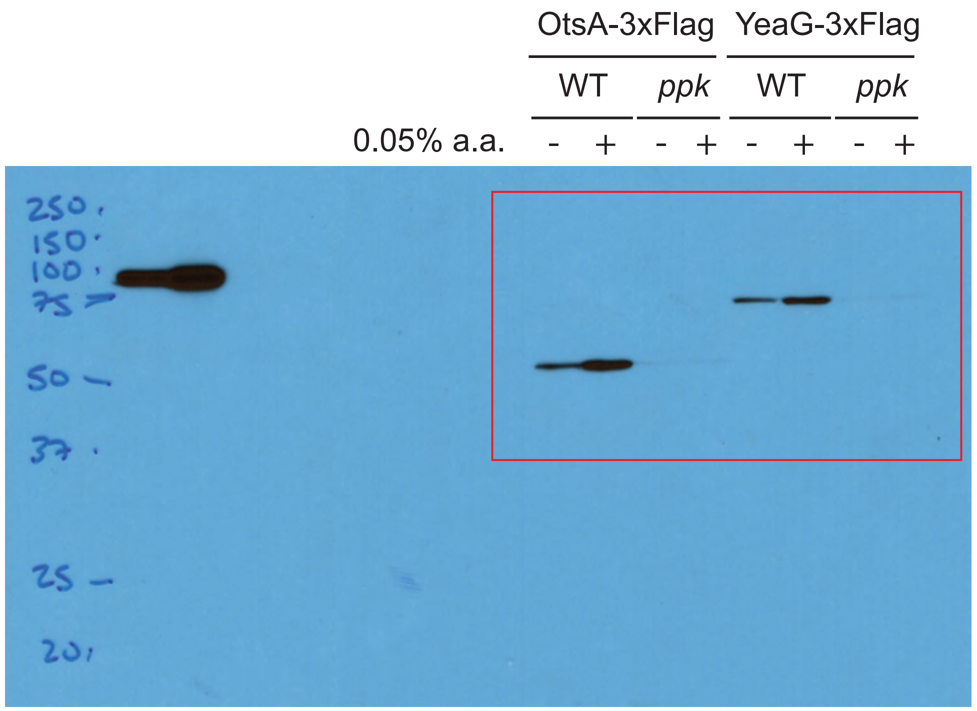

Exposure used for YbdL (Fig. 2E)

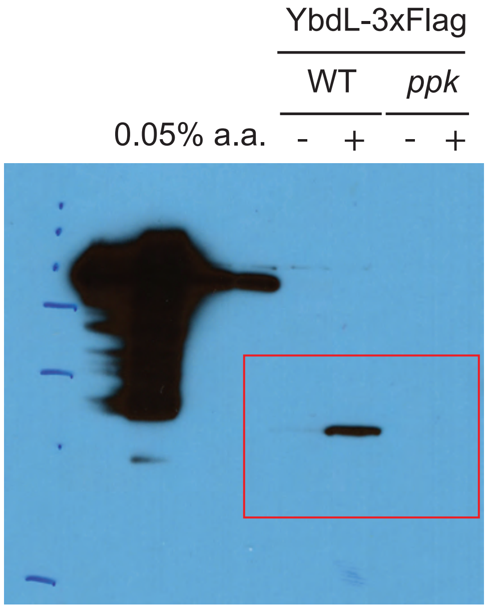

Corresponding ponceau for all exposures:

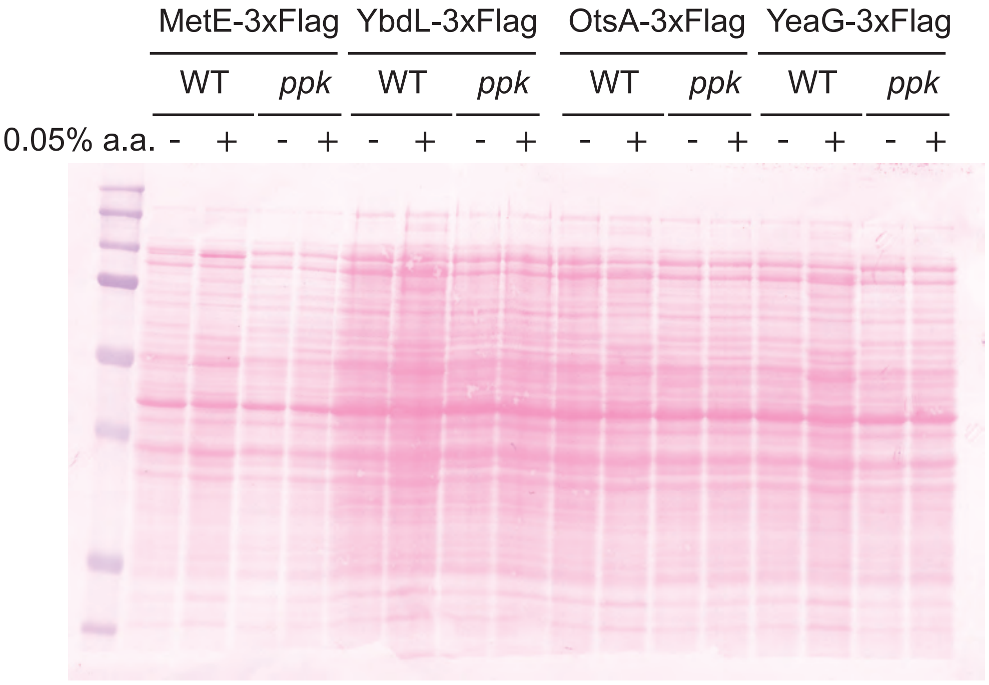

Raw data for Figure 3.

Blot used for EptA (Fig. 3B) and ArnT (Fig. 3D)

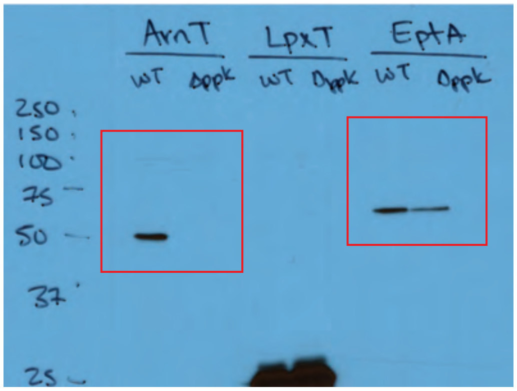

Corresponding ponceau for EptA (Fig. 3B) and ArnT (Fig. 3D)

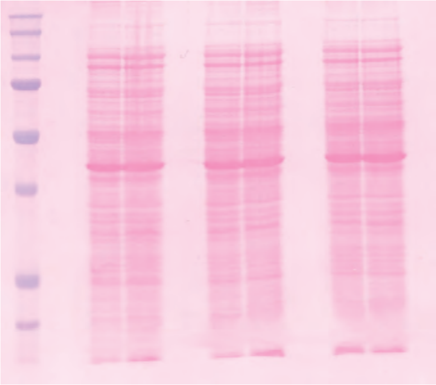

Blot used for ArnA (Fig. 3C)

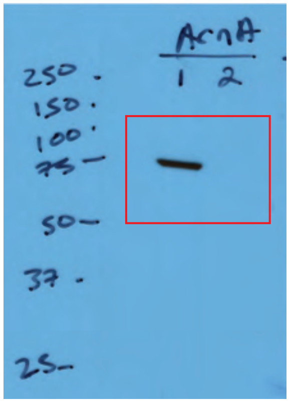

Corresponding ponceau for ArnA (Fig. 3C)

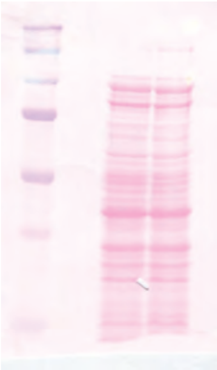

1 = WT
2 = Δppk mutant

Blot used for Fig. 3F

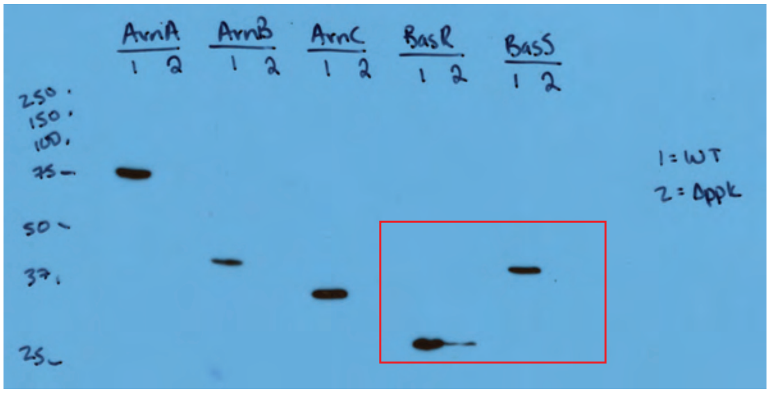

Corresponding ponceau for Fig. 3F

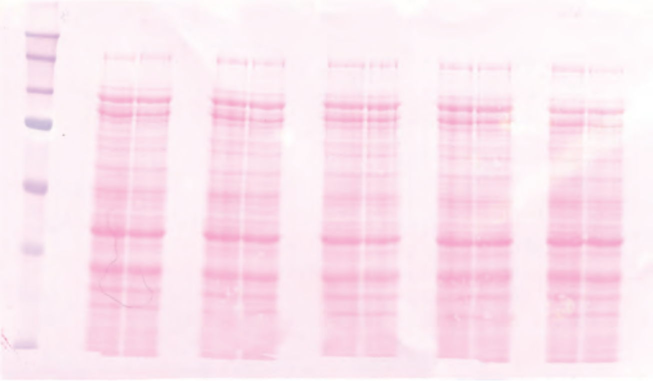

Blot used for Fig. 3H

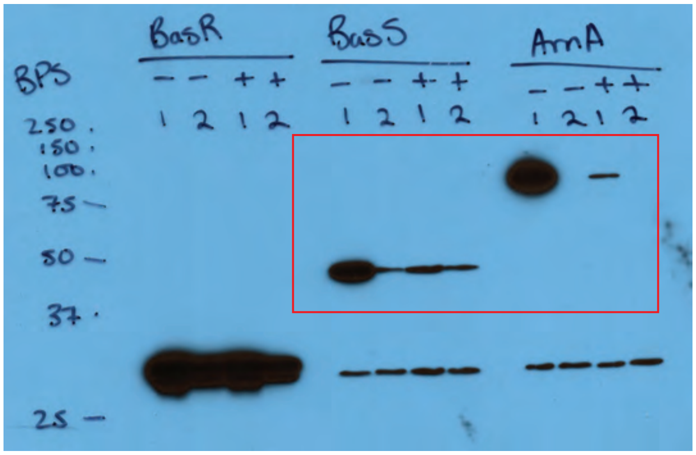

Corresponding ponceau for Fig. 3H

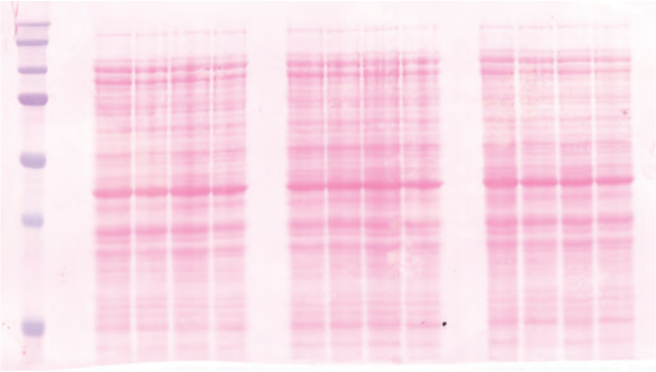

Blot used for Fig. 3I

ArnC-3xFlag

WT
Δ*ppk*
Δ*phoB*
Δ*phoB* Δ*ppk*

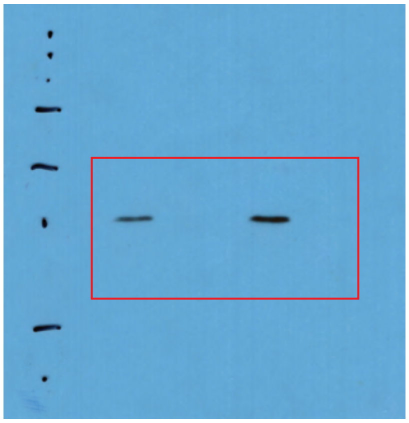

Corresponding ponceau for Fig. 3I

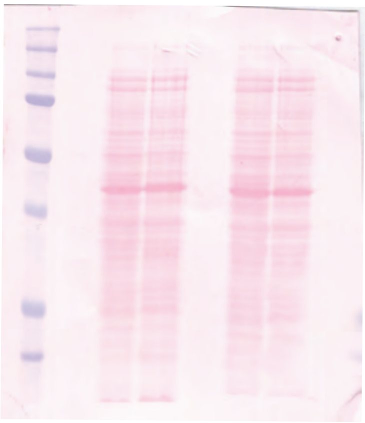

Raw data for Figure 4.

Raw image for lipid A analysis (Fig 4A).
To make the pEtN and L-Ara4N modifications clear linear brightness and contrast.
Linear adjustments were made evenly across the entire image shown (in Illustrator)..

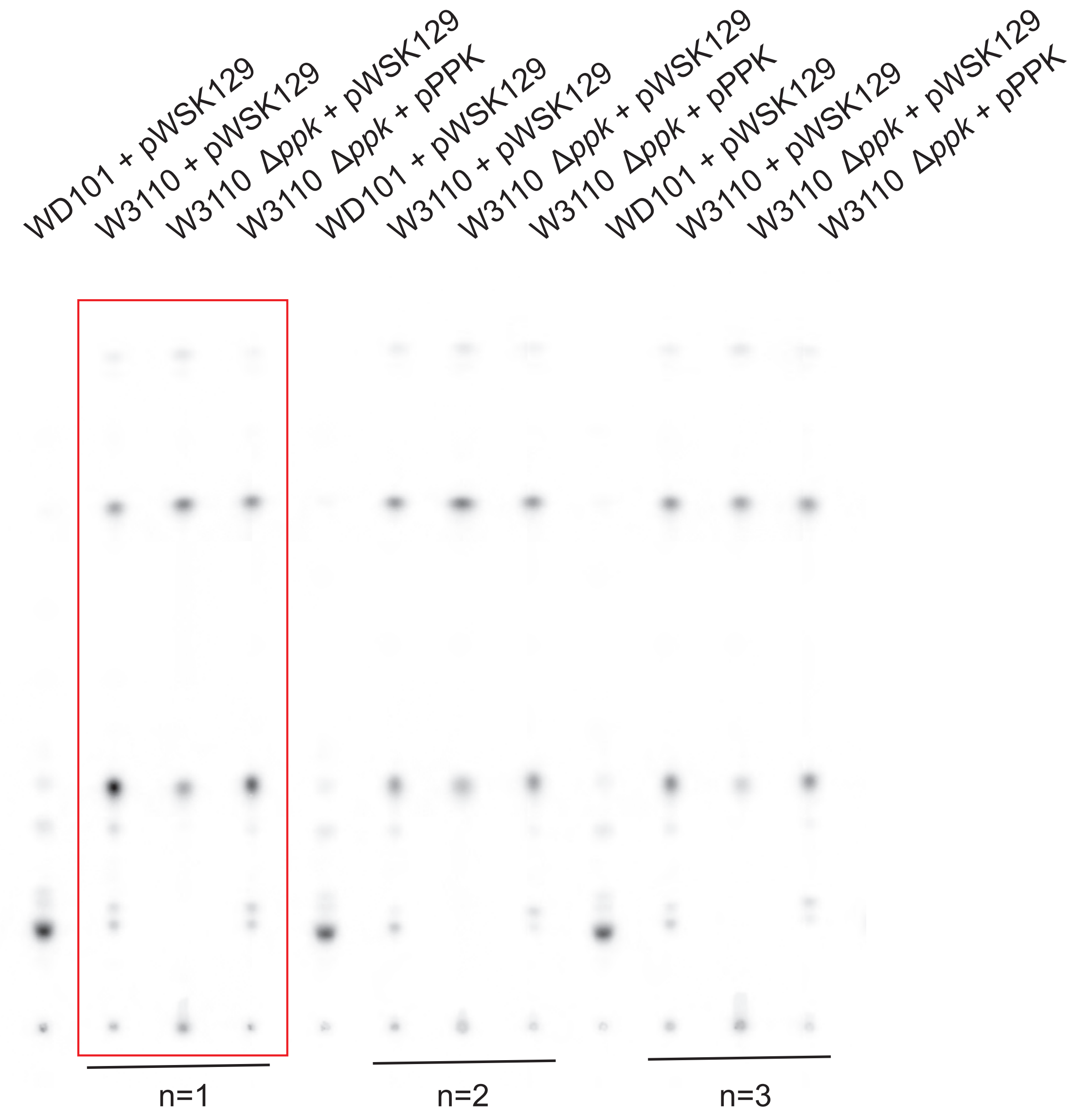

Spot test images used for Fig. 4B.

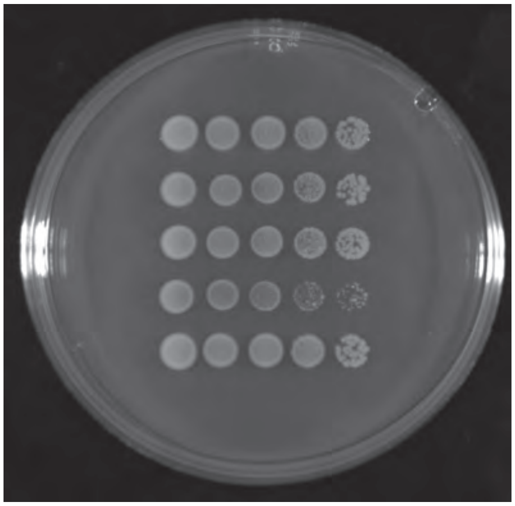

MOPS
0 ug/mL Polymyxin B

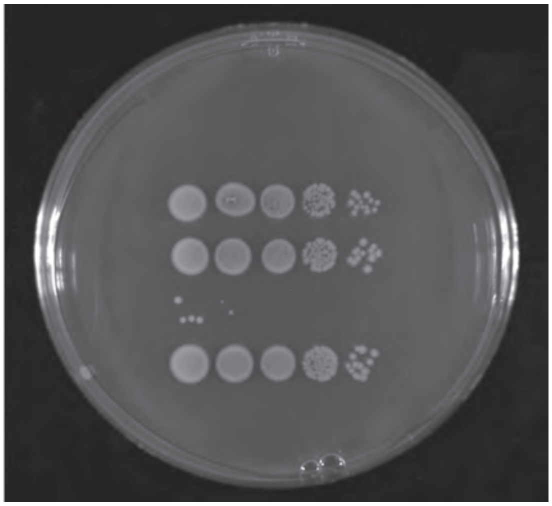

MOPS
1.5 ug/mL Polymyxin B

PolyP extraction gel used for Fig. 4C

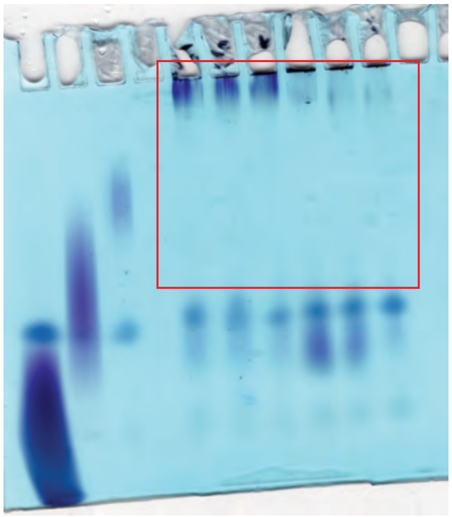

Blot used used for Fig. 4D

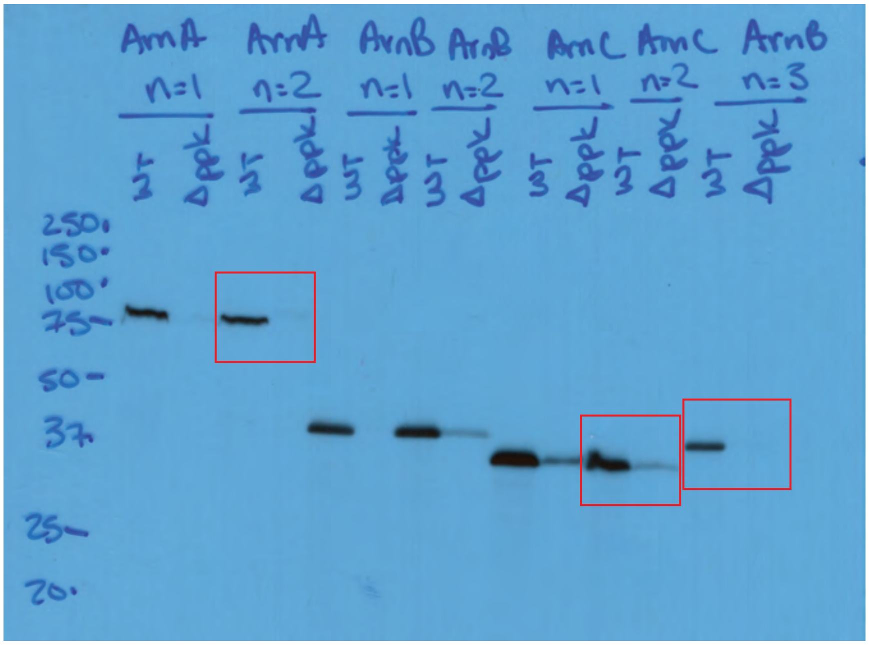

Corresponding ponceau for Fig. 4D

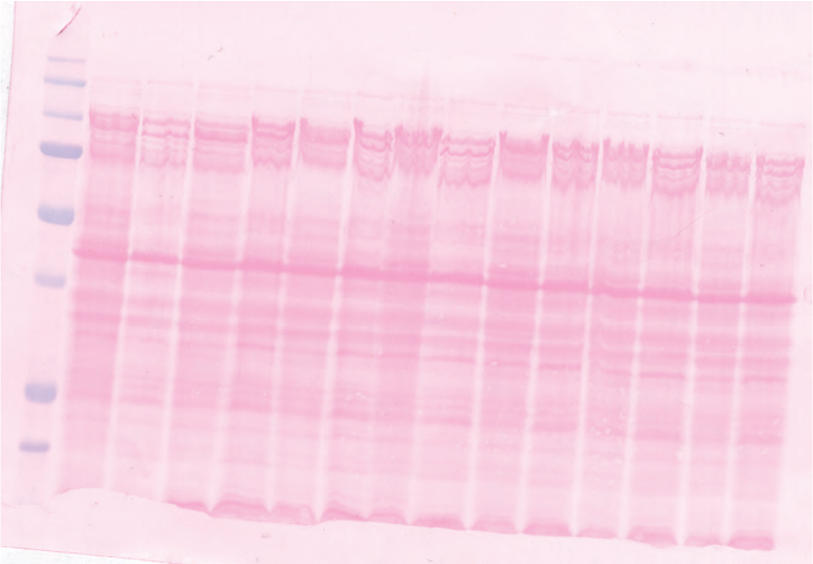

Raw data for Figure S1.

Uncropped scan of polyP extraction used for Figure S1.

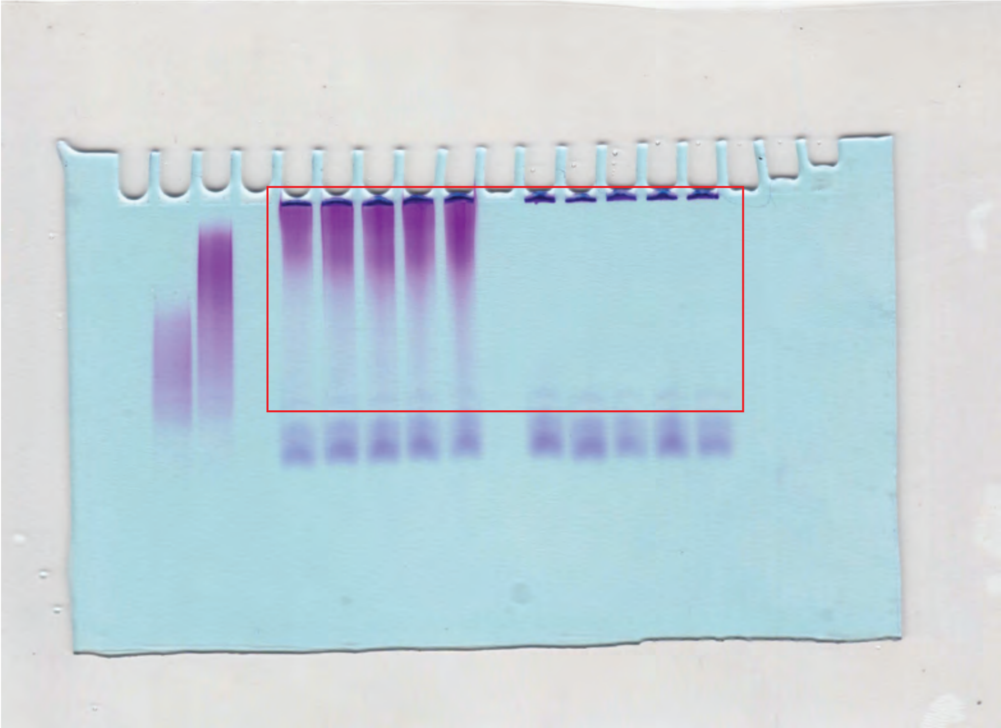

Raw data for Figure S2 western blots.

Blot used for Fig. S2

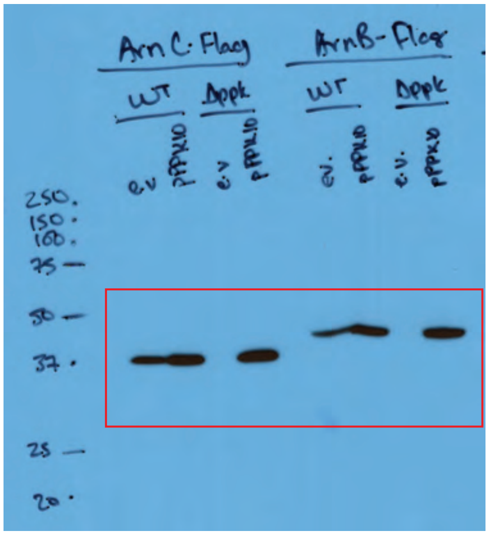

Corresponding ponceau for Fig. S2

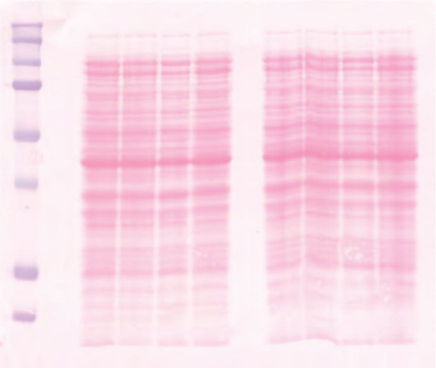

Raw data for Figure S3

Blot used for Fig. S3A

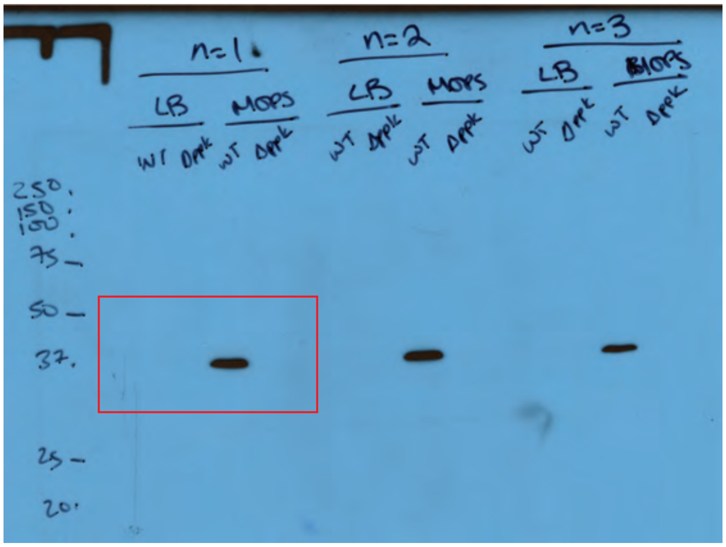

Ponceau used for Fig. S3A

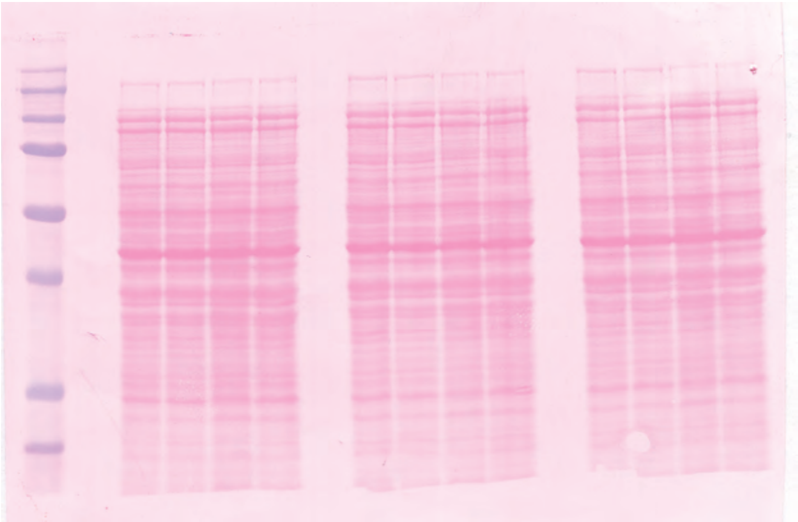

Blot used for Fig. S3B

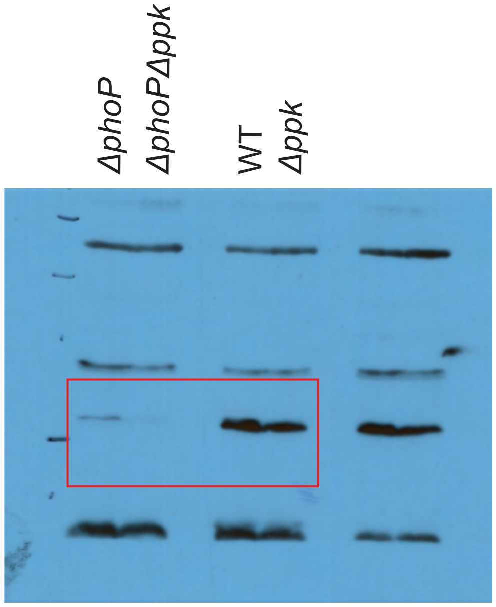

Blot used for Fig. S3C

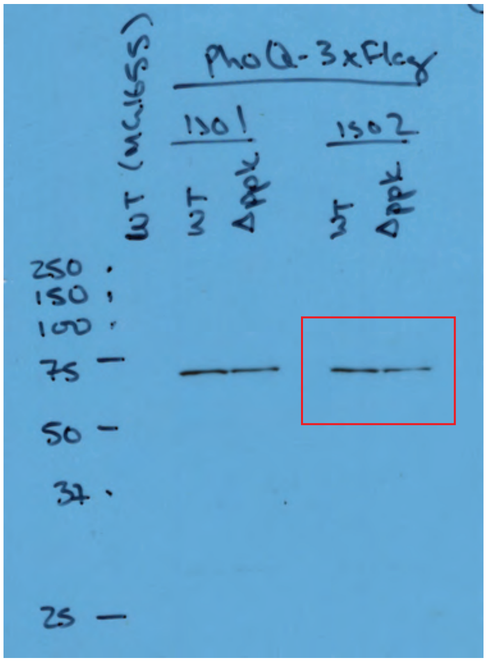

Ponceau used for Fig. S3B

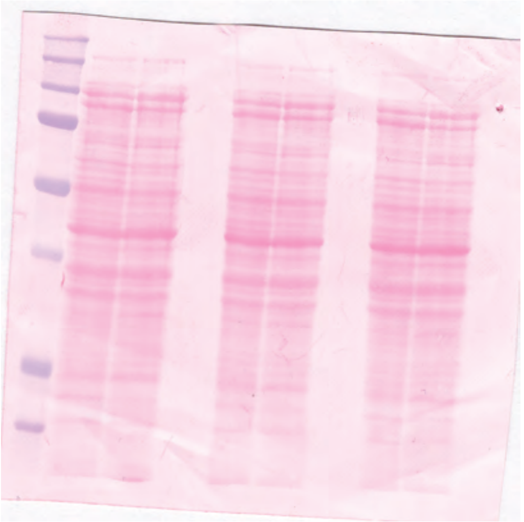

Ponceau used for Fig. S3C

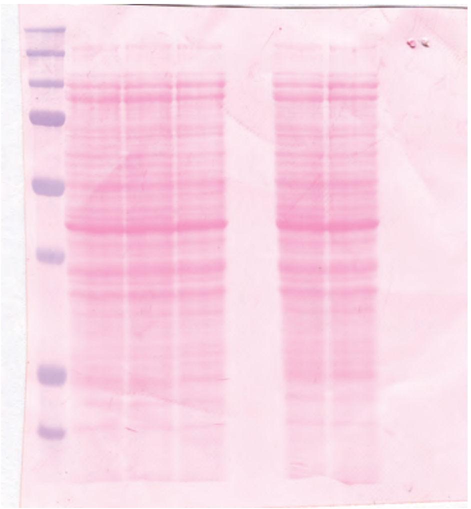

Light and dark exposures used for Fig. S3D

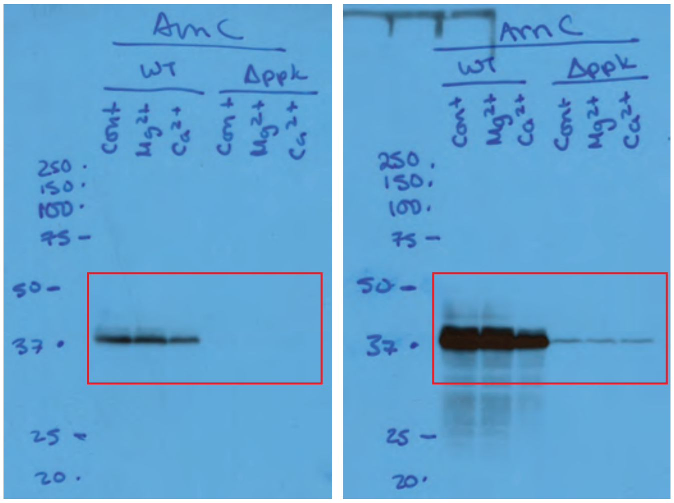

Ponceau used for Fig. S3D

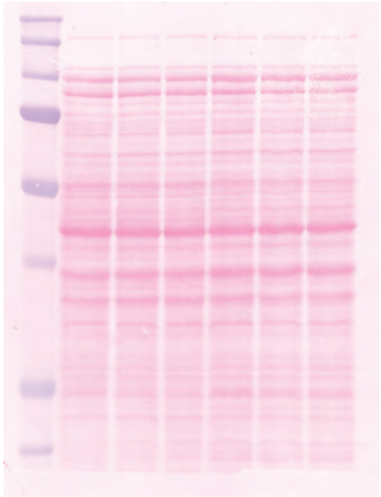

Blot used for Fig. S3E

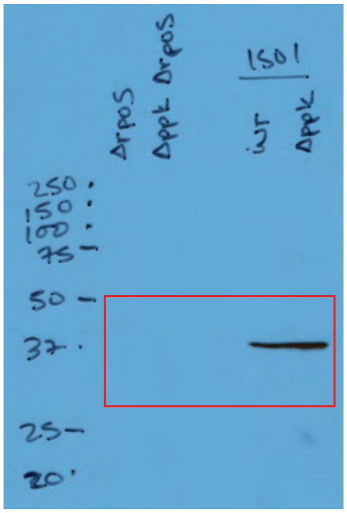

Ponceau used for Fig. S3E

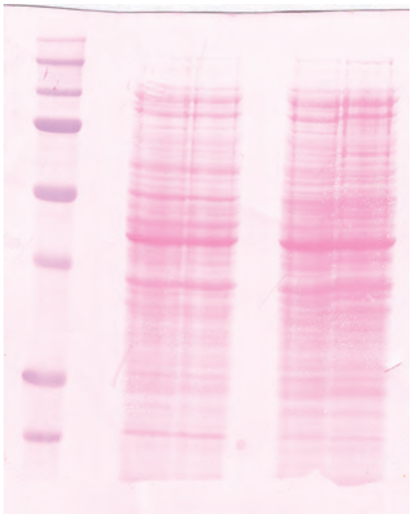

PolyP gel used for Fig. S3G

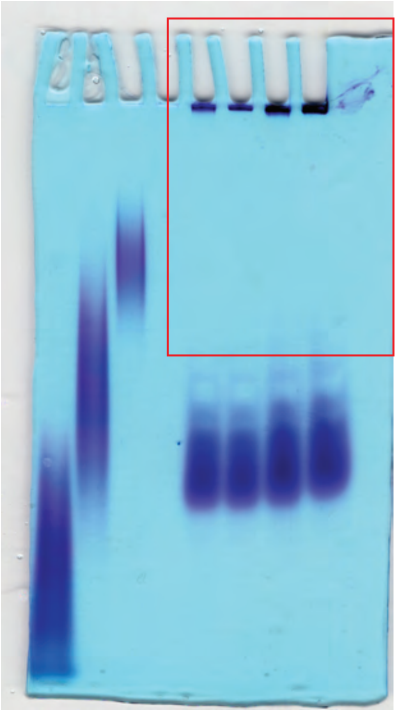

Blots used for Fig. S3F

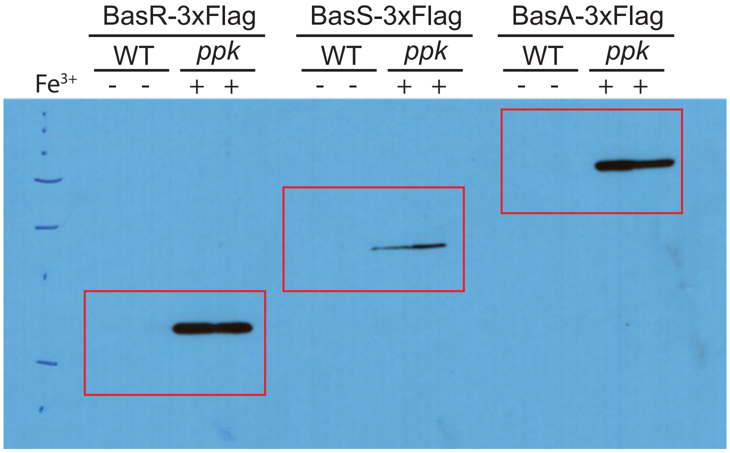

PolyP gel used for Fig. S3H

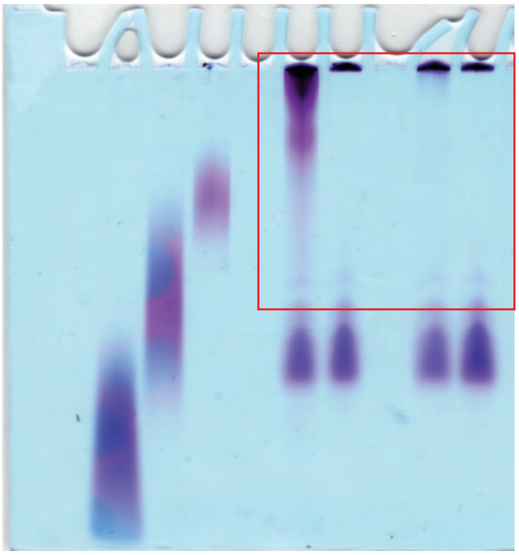

Ponceau used for Fig. S3F

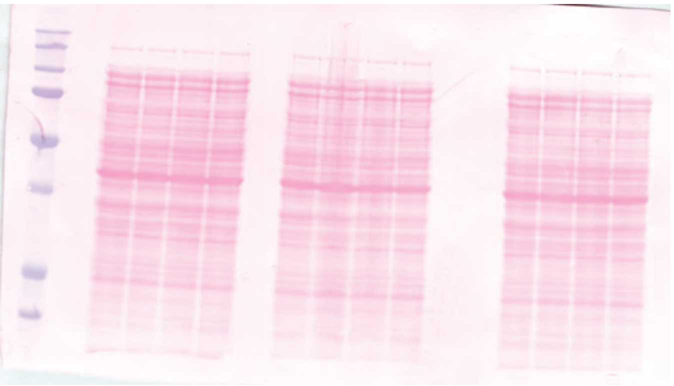

Raw data for Figure S4.

Blot used for Fig. S4A.

ArnA-3xFlag				ArnC-3xFlag			
W3110 WT	W3110 *Δppk*	WD101 WT	WD101 *Δppk*	W3110 WT	W3110 *Δppk*	WD101 WT	WD101 *Δppk*

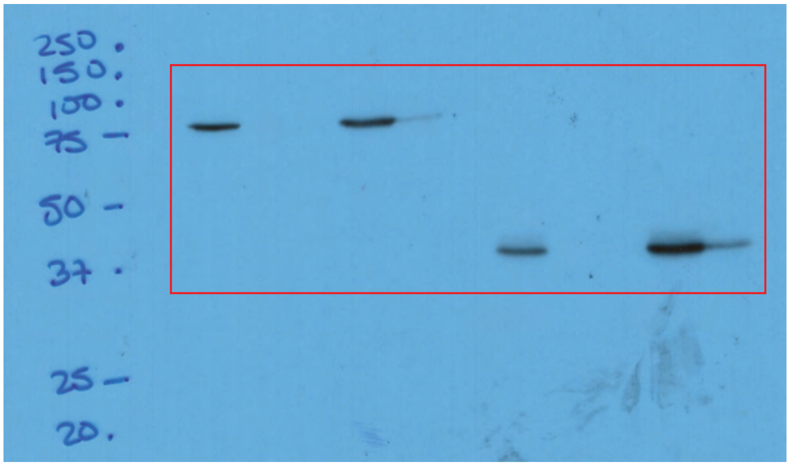

Corresponding ponceau for Fig. S4A.

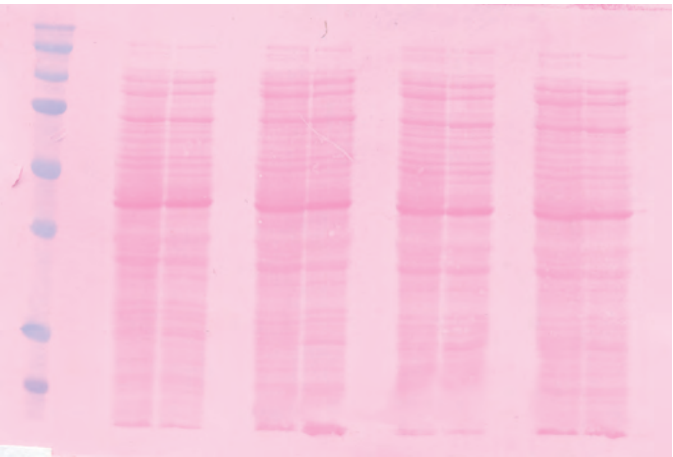

Blot used for Fig. S4B.

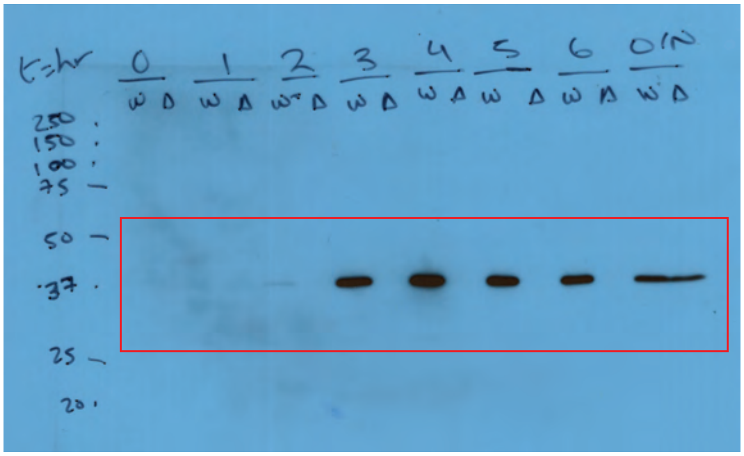

Corresponding ponceau for Fig. S4B.

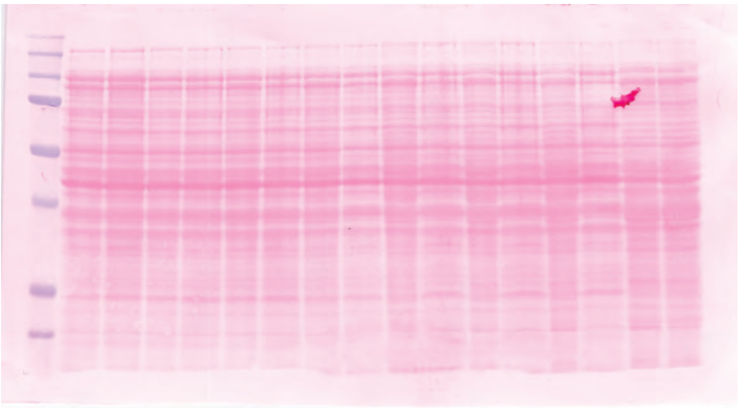

Spot test images used for Fig. S4C.

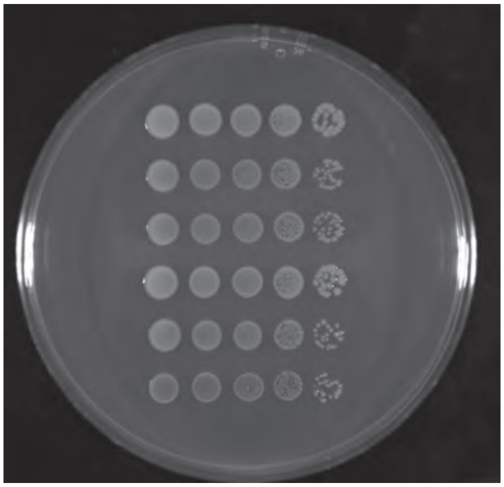

MOPS
0 ug/mL Polymyxin B

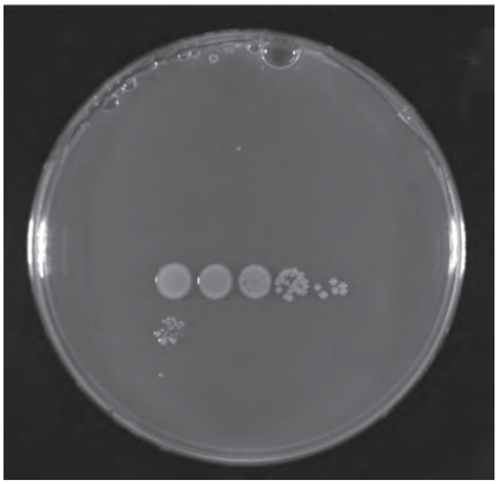

MOPS
1.5 ug/mL Polymyxin B

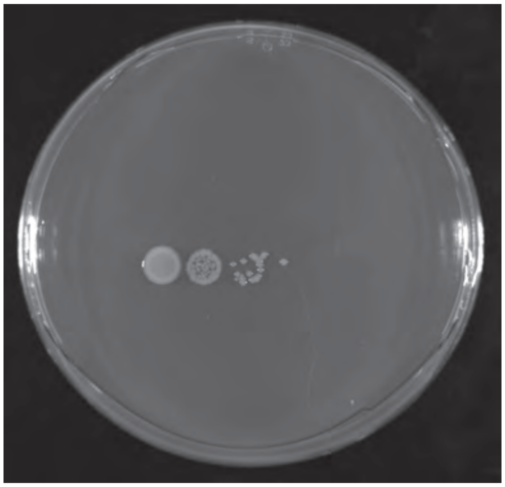

MOPS
2.5 ug/mL Polymyxin B

Spot test images used for Fig. S4D.

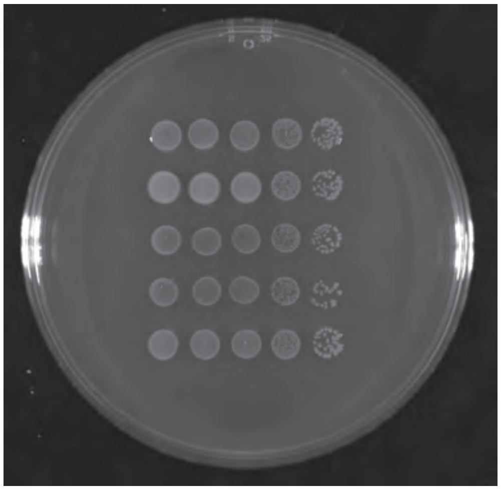

MOPS
0 ug/mL Polymyxin B

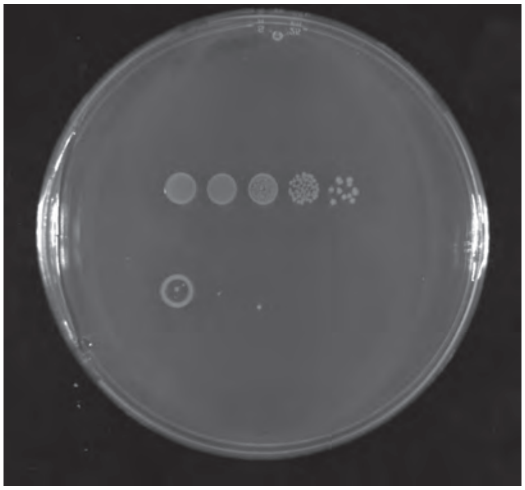

MOPS
1.5 ug/mL Polymyxin B

Raw data for Figure S6

Blot used for Fig. S6B

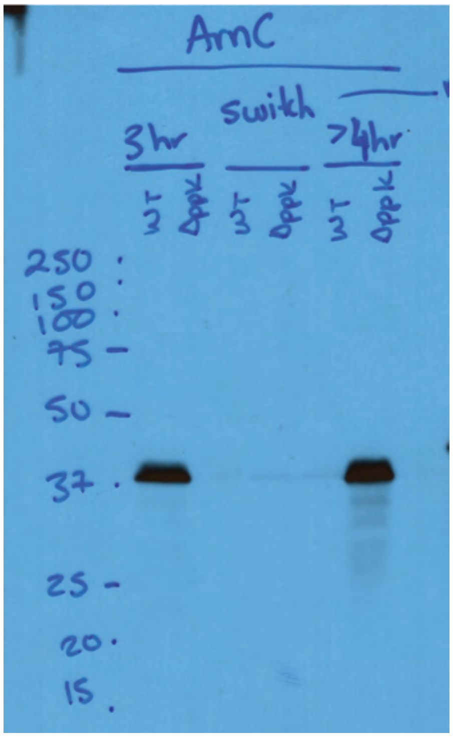

Corresponding ponceau for Fig. S6B

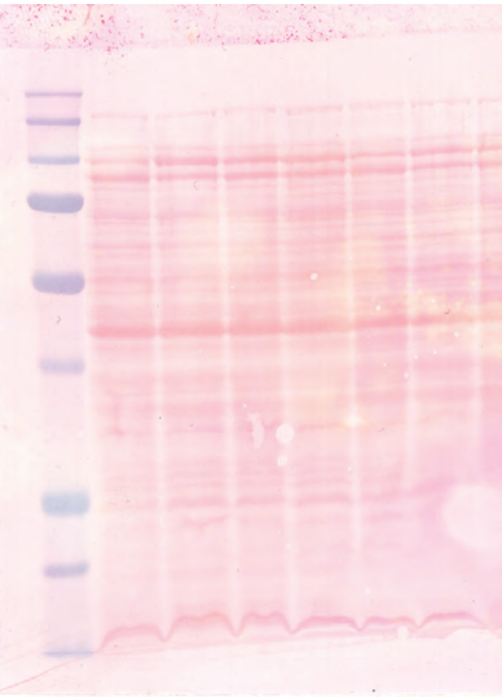

Supplement: S1 Raw Images — (PDF) [file pbio.3002558.s014.pdf]
